# A Trauma-Informed, Geospatially Aware, Just-in-Time Adaptive mHealth Intervention to Support Effective Coping Skills Among People Living With HIV in New Orleans: Development and Protocol for a Pilot Randomized Controlled Trial

**DOI:** 10.2196/47151

**Published:** 2023-10-24

**Authors:** Simone J Skeen, Stephanie Tokarz, Rayna E Gasik, Chelsea McGettigan Solano, Ethan A Smith, Momi Binaifer Sagoe, Lauryn V Hudson, Kara Steele, Katherine P Theall, Gretchen A Clum

**Affiliations:** 1 Department of Social, Behavioral, and Population Sciences School of Public Health and Tropical Medicine Tulane University New Orleans, LA United States; 2 Center for Community-Engaged Artificial Intelligence Tulane University New Orleans, LA United States; 3 Department of Psychology Hunter College City University of New York New York, NY United States; 4 Department of Epidemiology School of Public Health and Tropical Medicine Tulane University New Orleans, LA United States

**Keywords:** mobile health, mHealth, HIV, traumatic stress, posttraumatic growth, coping, geospatial, just-in-time adaptive intervention, JITAI, just-in-time, posttraumatic, medication adherence, mental well-being, viral suppression, coping, development, design, acceptability, feasibility, mobile phone

## Abstract

**Background:**

In 2020, Greater New Orleans, Louisiana, was home to 7048 people living with HIV—1083 per 100,000 residents, 2.85 times the US national rate. With Louisiana routinely ranked last in indexes of health equity, violent crime rates in Orleans Parish quintupling national averages, and in-care New Orleans people living with HIV surviving twice the US average of adverse childhood experiences, accessible, trauma-focused, evidence-based interventions (EBIs) for violence-affected people living with HIV are urgently needed.

**Objective:**

To meet this need, we adapted *Living in the Face of Trauma*, a well-established EBI tailored for people living with HIV, into *NOLA GEM*, a just-in-time adaptive mobile health (mHealth) intervention. This study aimed to culturally tailor and refine the NOLA GEM app and assess its acceptability; feasibility; and preliminary efficacy on care engagement, medication adherence, viral suppression, and mental well-being among in-care people living with HIV in Greater New Orleans.

**Methods:**

The development of NOLA GEM entailed identifying real-time tailoring variables via a geographic ecological momentary assessment (GEMA) study (n=49; aim 1) and place-based and user-centered tailoring, responsive to the unique cultural contexts of HIV survivorship in New Orleans, via formative interviews (n=12; aim 2). The iOS- and Android-enabled NOLA GEM app leverages twice-daily GEMA prompts to offer just-in-time, in-app recommendations for effective coping skills practice and app-delivered *Living in the Face of Trauma* session content. For aim 3, the pilot trial will enroll an analytic sample of 60 New Orleans people living with HIV individually randomized to parallel NOLA GEM (intervention) or GEMA-alone (control) arms at a 1:1 allocation for a 21-day period. Acceptability and feasibility will be assessed via enrollment, attrition, active daily use through paradata metrics, and prevalidated usability measures. At the postassessment time point, primary end points will be assessed via a range of well-validated, domain-specific scales. Care engagement and viral suppression will be assessed via past missed appointments and self-reported viral load at 30 and 90 days, respectively, and through well-demonstrated adherence self-efficacy measures.

**Results:**

Aims 1 and 2 have been achieved, NOLA GEM is in Beta, and all aim-3 methods have been reviewed and approved by the institutional review board of Tulane University. Recruitment was launched in July 2023, with a target date for follow-up assessment completion in December 2023.

**Conclusions:**

By leveraging user-centered development and embracing principles that elevate the lived expertise of New Orleans people living with HIV, mHealth-adapted EBIs can reflect community wisdom on posttraumatic resilience. Sustainable adoption of the NOLA GEM app and a promising early efficacy profile will support the feasibility of a future fully powered clinical trial and potential translation to new underserved settings in service of holistic survivorship and well-being of people living with HIV.

**Trial Registration:**

ClinicalTrials.gov NCT05784714; https://clinicaltrials.gov/ct2/show/NCT05784714

**International Registered Report Identifier (IRRID):**

PRR1-10.2196/47151

## Introduction

### Background

Trauma, violence, and survivorship are often closely intertwined for people living with HIV [1-4]. Among people living with HIV, posttraumatic stress disorder (PTSD) prevalence has been estimated at 28% worldwide [5]. Women living with HIV endure PTSD at rates as high as 35% (nearly 10 times the estimated national prevalence among adults in the United States) [5,6], with up to 97.1% reporting lifetime trauma histories in clinic-based samples [7], including cumulative adverse childhood experiences [8,9]. Early-life adversities are often compounded by syndemic food insecurity [10,11]; poverty [12]; and chronic prolonged exposure to relational, sexual [1,2,13,14], and community violence [15], particularly among Black people living with HIV, who are diagnosed at 22 to 33 times the rate of White American individuals [16]. Among seropositive young Black men who have sex with men enrolled in the Chicago-based Project nGage, which studied personalized social support as facilitator of antiretroviral therapy (ART) adherence, 40% had a close friend or relative die in violent circumstances; most had endured secondhand violence exposure—hearing gunshots (78%) and witnessing a physical assault (59%) [17]. Indeed, the syndemic effects of chronic violence exposure are often multiplicative, compounding, and disruptive to HIV continuum of care (CoC) maintenance, particularly among Black people living with HIV and men who have sex with men [18].

The daily toll of survivorship (regardless of formal PTSD diagnosis) is considerable for violence-affected people living with HIV [18]. Somatoform symptoms, including headaches, muscle ache, sexual discomfort, and fatigue, lacking “organic” etiology [19,20], can affect people living with HIV with trauma histories [5,21]. Continuous community violence, even endured vicariously, can represent a chronic environmental stressor, leading to self-isolation, collapse of social support networks [18], and persistent avoidance of trauma-related settings [1,5,18]; these can include clinical care sites for people living with HIV [1,4], with implications for dropout from the HIV CoC and reluctance to engage in clinic-based social support [8]. Approximately half (48% [22]) of people living with HIV in multisite samples show diagnosable substance use disorder (SUD), the foremost predictor of poor CoC retention [23]. SUD can represent a form of maladaptive coping, which is especially burdensome for violence-affected people living with HIV. In clinic-based samples, high PTSD symptom burden can increase the likelihood of opioid misuse (adjusted odds ratio 1.55, 95% CI 1.31-1.83); hazardous alcohol use (adjusted odds ratio 1.28, 95% CI 0.07-1.52) [24]; and, among New Orleans people living with HIV, membership in heavy-drinking alcohol use typologies [25]. Comorbid PTSD, depressive symptoms, and SUD interact to drive sexual transmission risk behaviors and derail CoC milestones [13,23], incurring risk of individual virologic rebound and immunologic dysfunction [26-28] and endangering population-level, treatment-as-prevention goals [29]. These entanglements of violence, traumatic stress, and biobehavioral sequelae suggest the need for closely tailored, trauma-informed interventions to support both proximal psychosocial and distal CoC goals for violence-affected people living with HIV remains acute.

Among the limited range of trialed interventions that target people living with HIV–specific violence exposure, trauma symptoms, and related sequelae [2,30] in service of CoC and adherence end points, *active, effective (also known as adaptive) coping* has emerged as a key mechanism of action able to mediate intraindividual pathways from lifetime, violence-induced traumatic stress to SUD and sexual risk [1,2,4] and facilitate posttraumatic growth [31,32]. The 15-session group intervention *Living in the Face of Trauma* (LIFT) is exemplary of such an approach [9,33]. LIFT, and the specific coping skills it aims to impart, is grounded in the cognitive theory of stress and coping (CTSC) [33,34]. The CTSC, in translation to an intervention model, recognizes 2 types of coping: “problem-focused” coping—strategies that aim to change or proactively manage a stressor (recognizing it as *changeable*)—and “emotion-focused” coping—strategies that aim to exert control over an emotional reaction to a stressor (acknowledging that it is *unchangeable*) [35]. Examples of the former include structured problem-solving skills, whereas the latter can include techniques such as cognitive restructuring, a technique to challenge and dismantle automatic thoughts such as dwelling on single negative details, which can undermine self-efficacy and self-esteem [33]. LIFT, notably, is not a PTSD treatment [33]. Among violence-affected people living with HIV, these adaptive coping strategies are meant to reduce the burden of posttraumatic stress, interrupting automatic recourse to maladaptive strategies such as hazardous alcohol use and, by extension, mitigating forward HIV transmission risk behaviors such as ART nonadherence and CoC dropout [8,13,23,33,34].

In an early randomized controlled trial (RCT) among mostly Black (68.6%) people living with HIV with histories of childhood sexual abuse, 40% of whom surpassed Diagnostic and Statistical Manual of Mental Disorders, Fourth Edition, diagnostic thresholds for PTSD, LIFT demonstrated clinically significant improvements in intrusive symptom burden [34] and reduction in avoidant coping styles and traumatic stress symptoms longitudinally [36]. A theoretically grounded, Centers for Disease Control and Prevention “Best Evidence”–designated intervention [37], LIFT has been adapted to meet the needs of South African women living with HIV [38] exhibiting exceedingly high (≤76.6%) sexual abuse prevalence, leading to statistically significant reductions in avoidance and hyperarousal symptoms of PTSD and improved ART adherence motivations at the 3-month follow-up in exploratory outcome analyses. However, a group session component was poorly attended, a challenge that the investigators attributed to participant concerns about privacy, stigma, and transportation logistics [39]. The preliminary short-term success of this adaptation, *Improving AIDS Care after Trauma*, points to the “innovation adaptability” of LIFT [40] and to the attunement of its core components to the needs of people living with HIV across high-incidence, resource-constrained settings.

Orleans and Jefferson Parishes, which constitute the New Orleans metropolitan area, Louisiana, were home to 7048 people living with HIV in 2020, a rate of 1083 per 100,000 population—2.85 times the US national rate [41]. With the second highest 2019 Gini coefficient (0.6) in the United States [42] and Louisiana routinely ranked last in national indexes of health care and income equality [43], the New Orleans 2019 murder rate (30.7 per 100,000) was 5 times the national average [44]. Contemporary findings from the *New Orleans Alcohol Use in HIV* study show that an in-care cohort of New Orleans people living with HIV survived an average of 3.5 adverse childhood experiences (vs 1.6 nationally) [45], with 21.2% obtaining high (>3/5) PTSD scores on the *Primary Care PTSD Screen for the Diagnostic and Statistical Manual of Mental Disorders, Fifth Edition (DSM-5)* [46]. Disparate exposure to high-density alcohol outlet environments is conducive to harmful SUD-consistent consumption patterns and depressive symptoms among New Orleans people living with HIV [45], reinforcing previous research implicating place-based drivers of CoC dropout [47,48]. In short, Greater New Orleans presents a setting in which syndemic HIV, traumatic stress, and violence exposure burden may be amenable to accessible coping skills interventions tailored for people living with HIV.

### Study Objectives

This study aims to develop, pilot, and refine *NOLA GEM*, a smartphone-delivered LIFT adaptation informed by within-person geographic ecological momentary assessment (GEMA) data on time-variant stressors and symptom profiles among violence-affected people living with HIV in New Orleans [49]. Mobile health (mHealth) and internet-delivered interventions that activate evidence-based, trauma-informed psychotherapies have shown promise in enacting clinically significant PTSD improvement [50,51], with a 2018 Cochrane review pointing toward opportunities to bolster the evidence base and calling for closer attention to mechanisms of change [52]. The benefits of mHealth in advancing CoC milestones are well established [53,54], although innovations in cultural and contextual tailoring are urgently needed to ensure sustainability [55-57]. These benefits can be augmented by in-app geospatial awareness, building on prior work that leverages GPS to (for example) alert mHealth users to the proximity of HIV and sexual and reproductive health clinics [58,59] or, in contrast, use real-time GPS coordinates to (1) describe routine activity paths and (2) apply this geospatial awareness at a hyperlocal level to address the potential of spatial-environmental stressors to evoke traumatic stress and drive CoC churn and, alternatively, identify restorative spaces to reduce stress. By developing NOLA GEM, we engage these opportunities to advance the field; meet the needs of New Orleans people living with HIV [41,45,46]; and address the nonlinear, holistic nature of traumatic stress [1,2,4,8] by delivering effective coping skills education and reinforcement in real time [33].

We will engage these objectives via the following specific aims:

1. *Aim 1*: conduct GEMA with violence-affected people living with HIV to (1) describe activity spaces associated with CoC and (2) assess potential mediated pathways through mood, mental health, substance use, trauma cognitions, and coping to inform novel intervention targets.
2. *Aim 2*: develop NOLA GEM, an mHealth LIFT adaptation, incorporating GEMA-informed mediators of well-being tailored for New Orleans people living with HIV.
3. *Aim 3*: test the feasibility, acceptability, and preliminary efficacy of the NOLA GEM app against GEMA alone on primary CoC (care engagement, ART adherence, and viral suppression) and secondary mental well-being end points.

Throughout, we use “NOLA GEM” to refer exclusively to the app that was developed in aim 2 to record GEMA data and deliver adapted LIFT intervention content.

## Methods

### Overview

NOLA GEM was developed through a multiphase National Institute on Alcohol Abuse and Alcoholism–funded pilot (R34AA028961) applying GEMA (*aim 1*)- and in-depth interview (*aim 2*)–derived insights to adapt LIFT to a just-in-time adaptive mHealth modality [60,61]. Aim 1 was launched in March 2021 and closed out in September 2021 (although aim 1 eventually recruited a sample of 89, owing to scheduling constraints, a subset of 49 informed intervention development). Aim-2 interviews were conducted in March 2022 and April 2022. This study protocol recounts the concurrent triangulated aim-1 and aim-2 analyses, their findings, and the consequent development of NOLA GEM and describes the design and conduct of the acceptability, feasibility, and preliminary efficacy (*aim 3*) trial. Throughout, we adhered to the SPIRIT (Standard Protocol Items: Recommendation for Interventional Trials) guidelines [62] as modified for pilot and feasibility trials [63].

### Aim 1: GEMA

#### Overview

A GEMA approach triangulates conventional ecological momentary assessment (EMA) methods, in which repeated assessments of participants’ cognitions, affect, behaviors, and biomarkers (eg, salivary cortisol [64]) are taken at multiple daily intervals, contextualized by GPS coordinates [49,65]. Through GEMA, recall bias is mitigated, and naturalistic spatiotemporal influences on individual health can be inferred [65]. It is particularly well suited for detecting context-specific environmental stressors and facilitators of individual resilience [66,67]. Using GEMA, aim 1 intended to describe *activity spaces*, the spatial configurations in which routine events occur with characteristic rhythms [68,69] associated with durable within-person patterns (both risk markers and risk buffers) of adherence and care among people living with HIV in New Orleans. Particular attention was paid to detecting potential mediated pathways via mood, mental health, substance use and misuse, trauma cognitions, coping skills, and domain-general self-efficacy variables, indicating the potential to operationalize these constructs as intervention touch points or proximal outcomes [70,71].

A small observational pilot of the GEMA method was reported by Theall et al [66]. Detailed methods and full (N=89) aim-1 GEMA findings are forthcoming.

#### Study Setting

As a nested, microlongitudinal study, aim 1 recruited and used the baseline and medical record data of a subset of participants already enrolled in the New Orleans area longitudinal *New Orleans Alcohol Use in HIV* cohort study [72,73]. This parent study recruited through the HIV outpatient clinic of a large university-affiliated medical center at which approximately 50% of New Orleans people living with HIV receive care.

#### Participants and Procedures

Inclusion criteria for the parent study (N=365) included age of ≥18 years, documented HIV serostatus, and being in care at an HIV specialty clinic; any patient experiencing an acute illness within the previous 6 weeks, acute intoxication, or pregnancy was excluded, with the option to participate once the then-acute medical issue was resolved. Enrolling a longitudinal cohort, the *New Orleans Alcohol Use in HIV* study’s primary aims are to advance clinically actionable knowledge on the biological mechanisms and socioenvironmental drivers of HIV- and alcohol-related comorbidities [72,73]. Details of the parent study protocol are available in Appendix 1 of the study by Welsh et al [72]. For the sake of aim-2 intervention development, aim 1 recruited people living with HIV (n=49; a limited subsample because of scheduling delays incurred during the recovery from 2021 Hurricane Ida) on ART who either self-reported a personal history of violence or lived in a high-violence neighborhood in the parent study. These “either/or” multilevel eligibility criteria were formulated pragmatically to ensure sufficient statistical power. History of violence criteria included reporting childhood physical or sexual abuse or physical assault as an adult or endorsing moderate to high stress because of neighborhood crime and family or relational violence. Living in a high-violence neighborhood was defined as the respondent’s residential Census tract having violent crime rates at or above the 75th percentile for violent crime rates for tracts within Orleans Parish. According to these criteria, 96% (47/49) of the sample indicated an individual personal history of violence exposure, with 4% (2/49) endorsing tract-level violence exposure exclusively. Parent study participants who met the inclusion criteria were contacted initially by phone after randomizing the order in which outreach was conducted. If interested, they were asked to schedule a 1-hour dedicated baseline appointment at which informed consent procedures were undertaken, a baseline assessment was conducted using REDCap (Research Electronic Data Capture; Vanderbilt University) [74], the daily diary EMA measures were reviewed for comprehension, and a GPS app was installed on participants’ smartphones. Eligible people living with HIV who did not possess a smartphone were provided one by study staff. Once enrolled, daily diary and geospatial tracking data were collected for each individual over a continuous 14-day daily diary survey period.

The 49 aim-1 participants whose GEMA responses informed intervention development recounted a range of lifetime violence exposures at baseline: 24 (49%) recounted childhood domestic violence, 18 (37%) recounted childhood sexual assault, 26 (53%) recounted physical assault in adulthood, 20 (41%) recounted current crime and violence exposures, and 9 (18%) recounted current intrafamilial violence. Screener responses suggested a range of potential mental health challenges: of the 49 participants, 13 (27%) had borderline or clinical PTSD as defined by the DSM-5, a total of 19 (39%) had borderline or clinical anxiety, and 12 (24%) had borderline or clinical depression. ART nonadherence was reported by 28 (57%). The broader sociodemographic attributes of aim-1 participants are presented in Table 1.

**Table 1:** Aim-1 participant (n=49) sociodemographic attributes.

Characteristics		Values
Age (years), mean (SD)		57.06 (9.59)
Years living with HIV, mean (SD)^a^		23.00 (9.41)
**Sex assigned at birth, n (%)^b^**		
	Female	16 (33)
	Male	33 (67)
**Racial and ethnic identity, n (%)**		
	Black or African American, non-Hispanic	40 (82)
	Native Hawaiian and Pacific Islander, non-Hispanic	1 (2)
	White non-Hispanic	5 (10)
	White Hispanic	2 (4)
**Sexual identity, n (%)**		
	Heterosexual or straight	28 (57)
	Gay or lesbian	11 (22)
	Bisexual	7 (14)
	Missing	3 (6)
**Household income (US $) in the past year at BL^c^, n (%)**		
	<20,000	39 (80)
	20,000-39,999	9 (18)
	40,000-59,999	0 (0)
	60,000-84,999	0 (0)
	≥85,000	1 (2)
**Housing at BL, n (%)**		
	Single-family dwelling	45 (92)
	HIV-specific group facility	1 (2)
	Homeless or shelter	3 (6)
Homeless (lifetime), n (%)		22 (45)
Incarcerated (lifetime), n (%)		25 (51)

^a^Computed by subtracting the year of HIV diagnosis in the medical records from the present year (2022); 13 participants lacked a recorded year of diagnosis.

^b^In line with the 2-step approach to ascertaining transgender identity in sociomedical research [75], a “current gender” item is included in the follow-up assessments presently underway.

^c^BL: baseline.

#### Daily Diaries

The daily diary measures, which operationalized the EMA aspect of aim 1, assessed a range of intrapsychic constructs, behaviors, and environmental stressors, combining brief prevalidated scales with novel study-specific measures. Participants were prompted by SMS text message and asked to complete the daily diary measures within a 3-hour window 3 times daily: 8 AM to 11 AM, 1 PM to 4 PM, and 7 PM to 10 PM Central Time Zone. Qualtrics (Qualtrics International, Inc) hosted the survey measures and administered the preprogrammed SMS text message prompts [76].

The daily diary measures for aim 1, with sample items, is shown in Multimedia Appendix 1.

#### Geospatial Tracking

At aim-1 baseline appointments, participants were guided through the installation of Actsoft Encore GPS-tracking app [77]. Throughout each individual’s 14-day daily diary survey period, Encore captured longitudinal and latitudinal coordinates at 1-minute intervals when moving and 1-hour intervals when stationary (for aim 3, the NOLA GEM app itself will deliver daily diaries and record geospatial trajectories). GPS data were stored on a password-protected cloud server. Participant GPS data were then transformed into activity paths at 50-, 100-, and 200-meter buffers using Python (Python Software Foundation) [78]. Environmental exposures were assessed through the creation of activity paths and interceptions with the 3 buffer sizes. Both static and dynamic environmental exposures were captured. Static data included, for example, annual rates per US Census tract level of exposure such as alcohol outlet density, community violent crime rate, poverty, and concentrated disadvantage. Dynamic data included time-stamped data linked to daily GPS points, including time-incident violent and nonviolent 9-1-1 calls to service data points and police stop-and-frisk calls. Activity path data were then aggregated to be merged with daily diary data for triangulation.

#### Aim-1 Formative Insights

In the course of exploratory and interim analyses of baseline and daily diary data, a range of environmental stressors with salience to the everyday lives of New Orleans area people living with HIV emerged. The study’s original objective was to understand how local spatial exposure and daily experiences may serve as determinants of traumatic stress or retraumatization connected to downstream maladaptive coping behaviors such as alcohol use and consequent patterns of ART nonadherence [79,80]. Our preliminary analyses did not find associations between outcomes related to alcohol use and daily mood across activity path–based spatial alcohol and violence exposure. These null results necessitated a departure from the original aim-1 goal of building generalizable place-based exposures as just-in-time adaptive intervention (JITAI) inputs into the NOLA GEM app. As such, tailoring variables, and the daily diary items through which they were captured in aim 1, were selected based on their overall prevalence at baseline, capturing past and cumulative experiences and associations with key outcomes within individuals on a given day among aim-1 participants. In recognition of the irreducibly idiographic nature of spatial traumatic stress determinants evidenced in this study, an individually customizable geofencing solution was built into the eventual NOLA GEM app (detailed in the *NOLA GEM App* section).

These stressors, with relevant statistics illustrating the rationale for their inclusion as JITAI tailoring variables, are provided in Table 2.

**Table 2:** NOLA GEM app just-in-time adaptive intervention tailoring variables—description and rationale.

Stressor	Rationale	Sample item or items^a^
Mental health challenges	51.22% endorsed “somewhat” or “very” stressful mental health challenges (past 30 days)^b^	In the past 30 days, how stressful were the following events? You can say “Not Applicable” if you did not experience this event. Worried about my mental health (not at all, somewhat, a lot)
Financial challenges	Within individuals on a given day, more financial stress positively predicted^c^: Mental health worries: β=1.12, SE 0.36 Individuals who had greater financial stress over 14 days also had greater^c^: PTSD^d^ symptom burden: β=1.4, SE 0.44 Pain symptom burden: β=2.6, SE 1.24	The next few questions ask about difficult events you might have experienced today. Please mark all that you experienced today: Financial stress Had a problem with money (yes, no) Worried about running out of food (yes, no) Worried about paying rent or getting evicted (yes, no)
Violence: direct	57.32% experienced IPV^e^ 31.71% experienced sexual assault (lifetime)^b^	IPV was measured using the Composite Abuse Scale (Revised)–Short Form [81]. The following are a couple of difficult or stressful things that sometimes happen to people either in relationships or not. These things can happen with someone you know, like a caregiver or partner, or someone you don’t know well like a stranger or acquaintance. Sexual assault: rape, attempted rape, made to perform any type of sexual act through force or threat of harm (never happened, happened but not in the past 12 months, happened in the past 12 months)
Violence: indirect	37.80% heard gunfire 17.07% experienced or witnessed police violence or intimidation 14.63% witnessed robbery, assault, or verbal threats (past 30 days)^b^	In the past 30 days, how stressful were the following events? You can say “Not Applicable” if you did not experience this event. Heard gunfire (not, somewhat, very stressful) Experienced or saw police violence or intimidation (not, somewhat, very stressful) Saw someone get robbed, injured, or threatened (not, somewhat, very stressful)
Violence: posttraumatic stress	>82.98% experienced ≥1 PTSD symptom (14-day aim-1 study period)c	How much were you bothered by avoiding thoughts, activities, or feelings about difficult life events that have happened to you? (not at all, a little bit, moderately, extremely; scale adapted from Erwin et al [82])
Social support deprivation	Within individuals on a given day, reporting no social support predicted^c^: Mental health worries: β=1.66, SE 0.58 Negative affect: β=.69, SE 0.23 Individuals who reported less social support over 14 days also reported more^c^: PTSD symptoms: β=10.74; SE 2.5	The next few questions ask about difficult events you might have experienced today. Please mark all that you experienced today (checklist): Didn’t feel supported by friends/family (yes, no)
Social support deprivation	Within individuals on a given day, reporting an argument predicted^c^: PTSD symptoms: β=.97, SE 0.31 Negative affect: β=.59, SE 0.21	The next few questions ask about difficult events you might have experienced today. Please mark all that you experienced today (checklist): Had an argument with a friend, family member, or romantic partner (yes, no)
Internalized HIV stigma	Within individuals on a given day, more negative feelings regarding HIV were positively associated with^c^: Mental health worries: β=1.05, SE 0.36 PTSD symptom burden: β=.25, SE 0.15 Negative affect: β=.31, SE 0.07^c^	Since last night/your last diary entry, how have you felt about your HIV status? Check all that apply (checklist): Ashamed (yes, no) Stressed (yes, no) Not as good as other people (yes, no)

^a^All aim-1 daily diary items are provided in Multimedia Appendix 1.

^b^Captured at baseline.

^c^Captured via daily diary.

^d^PTSD: posttraumatic stress disorder.

^e^IPV: intimate partner violence.

### Aim 2: Intervention Adaptation

#### Overview

Although GEMA can undergird rich, sophisticated statistical insights on the activity spaces of New Orleans people living with HIV, an overreliance on the “technocratic thrust” of decontextualized mathematical modeling risks overlooking the lived expertise of longtime New Orleanian survivors, with implications for external validity specifically [83,84] and epistemic justice, which is broadly defined as affording appropriate credibility to the knowledge- and sense-making practices of structurally marginalized groups such as people living with HIV [80,85]. As such, aim 2 recruited a subcohort of people living with HIV (n=12) enrolled in aim 1 to contribute their insights on stress, coping, and survivorship via in-depth interviews. This phase aimed to adapt LIFT in a manner that (1) activated the unique, uniquely powerful value propositions of mHealth [51,53,54,86] and (2) interlocked GEMA-derived intervention touch points with the core components of an extant evidence-based intervention (EBI) logic [33,34,36-39] while (3) recognizing the singular cultural and historical context of HIV survivorship in contemporary New Orleans. As such, aim 2 embraced principles of *design justice*, which call for a nonhierarchical, nonexploitative stance toward engaging the lived expertise of local communities [87] while leveraging the pragmatic, product-focused toolkit of *Lean User Experience*, which emphasizes an accelerated, iterative build-measure-learn approach to software development [88].

By invoking principles of epistemic and design justice, we aimed, in practice, to learn about and integrate community perspectives, needs, and especially strengths into the functionality of NOLA GEM [89]. This resulted in JITAI tailoring variables closely attuned to the needs of—and intervention content and proximal outcomes that reliably tap into the inductively derived strengths of—longtime violence-affected HIV survivors in New Orleans as “socially embedded, socially situated” community members [90]. In line with Jaworski et al [91] and Maestre et al [92], we synthesized mixed methods–derived insights in a manner calibrated to (1) appreciate local, culturally congruent nuances in our constructs of interest; (2) understand the precise conditions in which digital tools are used (and not used) among New Orleans people living with HIV; and (3) foreground coping focally and emotional well-being broadly as determinants of distal biomedical outcomes such as virologic control while calling on an extant EBI [33,34,36-39]. This approach is in line with core design justice principles, specifically design intentions committed to healing, sustainment, and empowerment; the catalyzation of people living with HIV end users’ lived expertise with formal design techniques; and, in particular, embracing “what is already working,” both via an adaptation of a demonstrated EBI and by incorporating aim-2 participants’ own drivers of survivorship and resilience while respecting users’ personal beliefs and local knowledge [87].

#### LIFT Intervention

Overarchingly, LIFT activates the CTSC, relying on that theory’s distinction between problem- and emotion-focused coping [33-35]. Through this lens, a range of adjunctive coping skillsets is taught, practiced, and discussed by group members in the course of a LIFT implementation in the field. These include breathing awareness and retraining, mindfulness meditation, journaling and distinguishing problem- versus emotion-focused and adaptive versus maladaptive coping styles, setting attainable personal goals, and surmounting common trauma cognitions (eg, all-or-nothing thinking). Theoretically grounded details of their precise trauma-informed mechanisms of action are provided by Sikkema et al [9,34,36] and in the *LIFT Manual* (hereafter, the *Manual*) [33]. The essentials of integrating each skillset into a LIFT participant’s daily life are reinforced through a range of skillset-specific handouts (eg, “Handout 4: Guidelines for Journal Writing”). The content of the NOLA GEM app was adapted by combining the *Manual*’s facilitator notes, sample (facilitator) scripts, and handouts [33]. In resituating the provision of this skill-building content from an in-person sequential group modality to a smartphone-delivered just-in-time modality, we envisioned use cases in which, for instance, the stress of hearing gunshots in the morning could be ameliorated via real-time access to breathing awareness or journaling (both emotion-focused coping skills per the CTSC [35]), interrupting the possibility of resorting to maladaptive coping strategies such as alcohol misuse.

#### JITAI Approach

A JITAI provides individually tailored intervention content precisely when needed, typically by tapping into the key affordances of smartphone-based models of delivery. Key elements of a JITAI include decision points, the time points at which the precise provision of intervention content is decided; tailoring variables, the data inputs that determine the nature of the intervention content offered; and decision rules, which embed the logics of tailoring variables at each prespecified decision point [60,61]. The end points of a JITAI logic are *proximal* (or short-term) outcomes, typically mediators of an intervention’s ultimate *distal* outcomes [60]. JITAIs have been reliably demonstrated to drive significant improvements across behavioral health domains in pretest-posttest trials and against waitlist, inert, and active controls [93].

#### Semistructured Interviews

To attain a balanced age distribution, aim 2 planned, initially, to recruit 5 to 10 participants from two studies: (1) the parent *New Orleans Alcohol Use in HIV* cohort (age range 18-65 years [72,73]), whose participants’ ages were concentrated in middle adulthood (median 50, IQR 41-56 years), and (2) the adolescent and young adult–focused Adolescent Medicine Trials Network for HIV/AIDS Interventions cohort (age range 12-24 years [94]; aim 2 planned to recruit participants aged ≥18 years exclusively). Ultimately, we were only able to enroll participants (n=12; mean age 57.83, SD 10.12 years; 58.33% Black and 75% male) from the parent longitudinal study [72]. Interviews ranged from 34 minutes, 31 seconds to 1 hour, 32 minutes, 18 seconds in length. Participants were given the option of a Zoom (Zoom Video Communications)-conducted [95] versus an in-person interview; 2 preferred Zoom. Adherent to the Tulane University Health Sciences Center COVID-19 control protocols then in place, all participants were required to bring proof of vaccination. Interviewers, who conducted the interviews in pairs, and participants remained masked at all times. Data collection proceeded subsequent to a verbal informed consent script.

The sociodemographic attributes of the aim-2 participants are shown in Table 3.

**Table 3:** Aim-2 participant (n=12) sociodemographic attributes.

Characteristics			Values
Age (years), mean (SD)			57.83 (10.12)
Years living with HIV, mean (SD)^a^			26 (7.93)
**Sex assigned at birth, n (%)^b^**			
	Female	3 (25)	
	Male	9 (75)	
**Racial and ethnic identity, n (%)**			
	Black or African American, non-Hispanic	7 (58)	
	Native Hawaiian and Pacific Islander, non-Hispanic	0 (0)	
	White non-Hispanic	4 (33)	
	White Hispanic	1 (8)	
**Sexual identity, n (%)**			
	Heterosexual or straight	5 (42)	
	Gay or lesbian	5 (42)	
	Bisexual	2 (17)	
**Household income (US $) in the past year at BL^c^, n (%)**			
	<20,000	8 (67)	
	20,000-39,999	2 (17)	
	40,000-59,999	1 (8)	
	60,000-84,999	0 (0)	
	≥85,000	1 (8)	
**Housing at BL, n (%)**			
	Single-family dwelling	9 (75)	
	HIV-specific group facility	2 (17)	
	Homeless or shelter	1 (8)	
Homeless (lifetime), n (%)			7 (58)
Incarcerated (lifetime), n (%)			6 (50)

^a^Computed by subtracting the year of HIV diagnosis in the medical records from the present year (2022); 2 participants lacked a recorded year of diagnosis.

^b^In line with the 2-step approach to ascertaining transgender identity in sociomedical research [75], a “current gender” item is included in the follow-up assessments presently underway.

^c^BL: baseline.

Interviews were semistructured in format and mixed methods interpretivist in orientation [83]. Initially, participants were prompted to designate how helpful they would find the skillsets offered by LIFT and a range of potential mHealth modalities (eg, SMS text messages or games) on a 4-point forced-choice Likert-type scale. The aim-2 sample was LIFT naive; the particular coping skillsets were introduced via brief interviewer-administered synopses (eg, “When managing daily stress related to life with HIV, how helpful would you find breathing retraining or finding different ways to breathe?”), with scripted in-depth definitions abstracted from the *Manual* available as needed (eg, “The way we breathe can affect the way we feel. Daily stress and anxiety can change the way we breathe and keep us feeling stressed. Breathing retraining describes ways we can adjust our breathing to help us relax when managing everyday stress”). Unstructured probes reflecting interviewees’ own language wherever possible elicited further detail on within-person patterns of successful coping grounded in the lived experiences of violence-affected people living with HIV in New Orleans. In addition, participants were asked to describe stress-inducing and reliably calming places and personal histories of violence, spirituality, relationships, drugs, and alcohol. A series of items grounded in design justice principles [87] prompted participants to reflect on the idiographic influences of their gender, ethnoracial and cultural heritage, and ability or disability on (1) their preferred stress management techniques and (2) the ways in which they interact with and selectively disengage from digital technologies. Each interview culminated in an adapted think-aloud exercise calling upon tenets of human-centered design [96,97] in which participants were reminded of the LIFT skillsets and mHealth modalities that they had previously considered “helpful” or “very helpful” and of particular stressors that they had characterized as “able to throw [their] whole day’s plans off track,” incite drug and alcohol misuse, or disrupt ART adherence. Aiming to encourage creative leaps [96] while eliciting context-specific JITAI dynamics rooted within the life worlds of New Orleans people living with HIV, participants were asked to narrativize precisely how their preferred LIFT skillsets would aid their coping through their most acute stressors, with impromptu probes (whenever possible) articulating details of their ideal mode of smartphone-based delivery. All interviews were audio recorded using a Sony ICD-PX470 digital voice recorder and transcribed verbatim.

#### Aim-2 Analysis

The qualitative analyses were subdivided into two phases, each with a dedicated intervention adaptation purpose:

1. A rapid inductive analysis dedicated to identifying *contender proximal outcomes*, a JITAI-specific construct that describes measurable potential mediators on the causal pathway to attaining *distal* (ie, our primary) outcomes.
2. A coding reliability thematic analysis dedicated to identifying survivorship and resilience themes, which would guide the adaptation of manualized LIFT worksheets into in-app NOLA GEM content responsive to the experiences of violence-affected New Orleanian people living with HIV. These themes were not operationalized as potential mediators but, rather, served to prioritize elements of the *Manual* to retain in the necessarily briefer NOLA GEM multimedia content.

The app development timeline necessitated this subdivision. Programming operationalized contender proximal outcomes as “SkillCheck” quizzes (described later in this paragraph) was an early, urgent development milestone, whereas in-app content adaptation could occur on a more generous, investigator-initiated schedule. The analytic procedure of the first phase of aim 2 relied, first, on a variant of rigorous and accelerated data reduction, a rapid matrix analysis technique [98]. Coders (n=7) condensed each interview into its richest, most salient excerpts using matrices in Microsoft Excel (Microsoft Corp) [99]. Independently, each coder inductively developed first-cycle (“open” in grounded theory terminology) descriptive, process, and in vivo codes [100]. Concurrently, each coder, consistent with a causation coding approach [100], identified contender proximal JITAI outcomes—inductively derived potential mediators of the NOLA GEM intervention’s predetermined distal outcomes [60,61]. The first author (SJS), in collaboration with LVH, synthesized these initial codes and contender proximal outcomes into second-cycle (“axial”) codes. These contender proximal outcomes were then preliminarily operationalized as “SkillCheck” quizzes, which would be provided to end users upon completion of prespecified units of intervention content (a process that is detailed in the *Intervention Design* section).

Subsequently, in the analysis in the second phase of aim 2, the second-cycle coding schema and complete interview data set were migrated to Dedoose (SocioCultural Research Consultants) [101]. In Dedoose, 3 coders, accompanied by the first author (SJS), pilot coded randomly selected full transcripts masked to each other’s coding applications, resolved coding discrepancies, and refined the final codebook in conference until a pooled intercoder reliability of κ=0.78 was achieved [102]. After obtaining acceptable intercoder reliability, the balance of transcripts was coded independently (KS, LVH, and MBS).

#### Aim-2 Formative Insights

A total of 10 contender proximal outcomes were detected through the rapid matrix analyses in the first phase of aim 2. These constructs and the SkillCheck items used to operationalize them as potential mediators are provided in Textbox 1.

Building on the insights from the first phase of aim 2, refined survivorship and resilience themes, with 2 nested subthemes, were detected across the inductive analyses in the second phase of aim 2. Methodologically, we generated second-cycle pattern codes, formatted as process codes, which rely on gerunds to (consistent with dynamic JITAI logics [60]) “imply actions intertwined with the dynamics of time” [100]. The complete set of themes, subthemes, definitions, and illustrative excerpts is provided in Table 4.

**Formative results of the first phase of aim 2—proximal outcomes and SkillChecks. All response options were 4-point forced-choice Likert scales (“strongly agree” to “strongly disagree”) in format. Although the phrase Please rate your agreement with the following statement recurs in each SkillCheck, we omitted it from subsequent textbox examples to avoid needless duplication.**

**Strengthened grounding skills**

- “Grounding” refers to focusing on our own bodies, how they occupy time and space, to reclaim our minds from unpleasant thoughts or feelings.
- *Please rate your agreement with the following statement*: This skill helped me: *build grounding into my everyday life*.

**Strengthened healthy routines**

- Healthy routines that reflect our authentic selves, interests, and practical wisdom can keep our minds occupied and self-care needs met.
- This skill helped me: *build healthy routines into my everyday life*.

**Strengthened positive social bonds**

- Having a few go-to people in our lives ready to listen and support us through a rough day is essential to our well-being.
- This skill helped me: *take steps toward building a supportive social circle into my everyday life*.

**Strengthened awareness of spatial triggers**

- Certain parts of the city—certain neighborhoods, corners, bars, or parks—can cause sudden painful memories or unpleasant feelings.
- This skill helped me: *think through certain spaces and places that can trigger my stress*.

**Strengthened redirect skills**

- “Redirecting” refers to recognizing the unhelpful or hurtful places where our thoughts may be taking us and conscientiously steering our minds in a more meaningful, productive direction.
- This skill helped me: *redirect away from negative coping strategies that arise in everyday life*.

**Strengthened insights on self-management capacity**

- Being honest with ourselves about the stressful situations that we can handle versus the situations we cannot keeps us out of situations that may compromise healthy decision-making.
- This skill helped me: *better understand the situations I can handle versus those I cannot*.

**Strengthened comfort with survivorship disclosures**

- It can take great strength to disclose our HIV survivorship, even if it emerges as a source of inner strength or a point of fellowship.
- This skill helped me: *ease stress and build my comfort around disclosing my HIV survivorship*.

**Strengthened help-seeking capacity**

- It can take self-honesty to admit when we need help sometimes, and reaching out to others when in need can be even harder.
- This skill helped me: *build my comfort in reaching out to others when I need help*.

**Strengthened forgiveness skills**

- It can take wisdom to let go of resentment, forgiving ourselves and others for past decisions that may have been hurtful or mistaken.
- This skill helped me: *build my ability to forgive myself and others*.

**Strengthened insights on spirituality and well-being**

- Deeper understandings of the mind, the soul, spirituality, and meditation often guide us away from unpleasant thoughts and feelings whether we consider ourselves religious or not.
- This skill helped me: *connect my inner life with my own day-to-day well-being*.

**Table 4:** Formative results of the second phase of aim 2—themes, definitions, and illustrative excerpts.

Theme	Definition	Example^a^
Accepting and adapting	Captures any instances in which participants describe accepting difficult or nonoptimal life circumstances, typically through processes of self-reflection or recognition of the limits of control over one’s life and its trajectory	“I’m the kind of person that can adapt to really anything. I would have maybe a little hissy fit at first, but I do cope with change well. And I just move on because that’s really all you can do in life.” [Participant M; Black male individual^b^; aged 42 years^c^]
Accepting and adapting (*self-reflecting*^d^)	Captures specific, explicit mentions of the self-reflective processes that facilitate accepting and adapting	“I have made some mistakes, but that’s history. They’ve been paid for, squashed. But you can reflect on that. It’s like, ‘Mm-hmm [affirmative].’ Just, you move on. Be happy about what you do, where you’re at. That’s the way I look at it.” [Participant R; White male individual; aged 63 years]
Affirming shared experience	Subsumes any mentions of forging social bonds regarding shared histories or experiences in common, often emphasizing learning and problem-solving	“Sometimes you just want to be by yourself and think things out. But sometimes, it’s also very helpful because you talk with somebody and you’re realizing, they have the same situation, and they do something that might help them and you’re open to try something else and maybe might help you.” [Participant H; White female individual; aged 54 years]
Cultivating spirituality	Captures any mention of the role of self-evolved, personal, idiographic modes of spirituality as preferred or relied-upon modes of coping; spiritual aspect must be explicit	“Well, I pray all the time. Not all the time, but frequently all day long. Just to stay calm. Help me stay calm. Not that I’m stressed out much, but [prayer] help me not get a tone.” [Participant F; White male individual; aged 65 years]
Embracing routine	Subsumes mentions of carrying out repeated, cadenced tasks or rituals to occupy the mind and body, typically emphasizing the stress mitigation aspect thereof	“I get up every morning and I clean my whole neighborhood stores up, the laundromat. They don’t pay me. I just walk and do it. And they’ve just been a routine all my life.” [Participant U; Black female individual; aged 70 years]
Enacting boundaries	Subsumes acknowledgments that restricting or eliminating contact with select individuals is protective of emotional, spiritual, and physical well-being; relational-interpersonal aspect must be explicit	“Just the idea of waking up being among the living, had something to do to pay attention other than being ’round people with bad influence, making you do something that you regret doing, or make your regret doing something they want you to do [to] satisfy them...why should I make myself miserable just to be your friend, knowing I’m going to pay for it?” [Participant I; Black male individual; aged 58 years]
Finding fellowship	Captures any mention of community as continuity and community as facilitating coping; includes faith communities, organized religion, and social bonds formed therein	“The rest of my relationships and the relationships in my life, they add bits and pieces to help my mental health basically, like talking to friends and when I need to vent, venting to my sisters...Like, ‘Hey, I got an issue. Hey, how would you do this, or let me bounce this off of you so I can understand where I’m coming from,’ or something like that.” [Participant Z; Black male individual; aged 39 years]
Regulating impulses	Subsumes mentions of conscientiously recognizing, confronting, minimizing, or extinguishing impulsive urges and behaviors	“I’ve experienced violence twice. I witnessed it on somebody else, and I made cognitive decisions that my partner is not going to have to deal with what my friend was dealing with his partner. And my coping mechanism is silence. Just silence. I just, rather than pop off and say things you regret later, I’ll just shut down.” [Participant E; White male individual; aged 67 years]
Regulating impulses (*rejecting substances*)	Subsumes instances of impulse regulation specifically, explicitly related to overcoming hazardous alcohol and drug use behaviors	“Because I used to use drugs about four years ago. Alcohol too. When I got rid of that, because when I got into my spirituals, I took away the alcohol and the desire for drug.” [Participant O; Black male individual; aged 60 years]
Self-soothing	Captures any instances in which healthy, adaptive activities chosen or routinely undertaken to minimize stress and exposure to stressors are described	“Pray. Little deep breaths, calm myself down, talk to myself ‘Calm down, don’t don’t let it get to you.’ Give myself my own little pep talk. Calm down, whatever. And just go and hang out with my grandkids and that’s destressed right there.” [Participant H; White female individual; aged 54 years]
Staying situationally aware	Captures any mention of vigilance with regard to one’s surroundings, often emphasizing the potential for violence (inclusive of law enforcement–enacted violence); spatial aspect must be explicit	“Well, [participating in GEMA] brought me to the point to be more observative...that really made me pay attention. Is you in drug free zone? Is it easy to get alcohol? Is it easy to get drug? And when I read those things, that made me alert not to go to those places because I know if I go around some of these places...I smell trouble.” [Participant I; Black male individual; aged 58 years]

^a^Excerpts have been edited lightly for conciseness.

^b^Assigned sex.

^c^Age at time of interview.

^d^Italicization denotes subthemes.

#### Intervention Design

Lean user experience (UX) recognizes the challenge of user-centered outcomes losing focus over the course of complex, multistakeholder technology development cycles—a challenge that is intensified when faced with extant, theoretically grounded EBI logics [33,34]. As such, we called on Lean UX techniques to integrate the full gamut of formative insights cultivated across aims 1 and 2, counteracting the (interrelated) possibilities of stakeholder misalignment or theoretical fragmentation [103] (as an added strength of this approach, Lean UX exists in tandem with the Agile workflows adopted by most contemporary software developers [88]).

**Figure 1:**
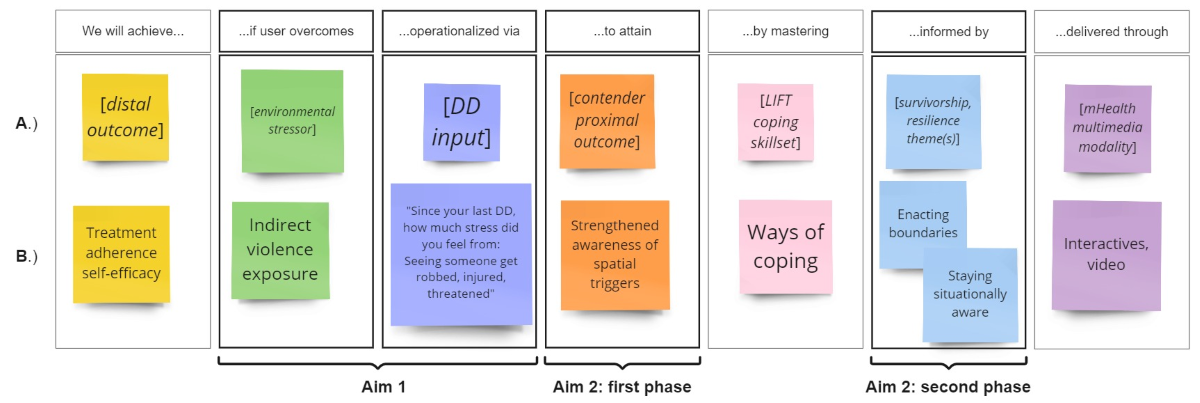
(A) Mocked-up feature hypothesis chart displaying column heads representing each element of a theoretically grounded, culturally tailored just-in-time adaptive intervention (JITAI) and (B) a sample JITAI logic tying together formative insights derived from aims 1 and 2. The format was adapted from Gothelf and Seiden, and the graphic was created in Miro. DD: daily diary; LIFT: Living in the Face of Trauma; mHealth: mobile health.

Specifically, using the Miro whiteboarding platform [104], we assembled a feature hypothesis chart and conducted a design workshop in which the formative research undertaken to date was organized according to a JITAI-consistent intervention logic [60,61,91]. *Hypothesis* in this context refers to an easily communicable single-sentence association between a user outcome; a software feature; and (in terms by Mohr et al [105]) the usage aims and elements of a behavioral intervention technology logic, whereby each element is positioned as a column header in the feature hypothesis chart. The workshop, facilitated by the first author (SJS) in June 2022, formulated feature hypotheses accordingly: “We will achieve [*distal outcome*] if user can overcome [*environmental stressor* (derived from Aim I descriptives)], operationalized via [*daily diary input* (selected from among Aim I measures)], to attain [*contender proximal outcome* (derived from aim-2 rapid matrix analyses)], by mastering [*LIFT coping skillset* (abstracted from the *Manual*)], in a manner informed by [*survivorship and resilience themes* (derived from Aim IIb thematic analysis)], delivered through [*mHealth multimedia modality*].” All faculty investigators and interviewers (serving as end-user advocates) were present. A mocked-up feature hypothesis chart is shown in Figure 1 [88,104].

**Figure 2:**
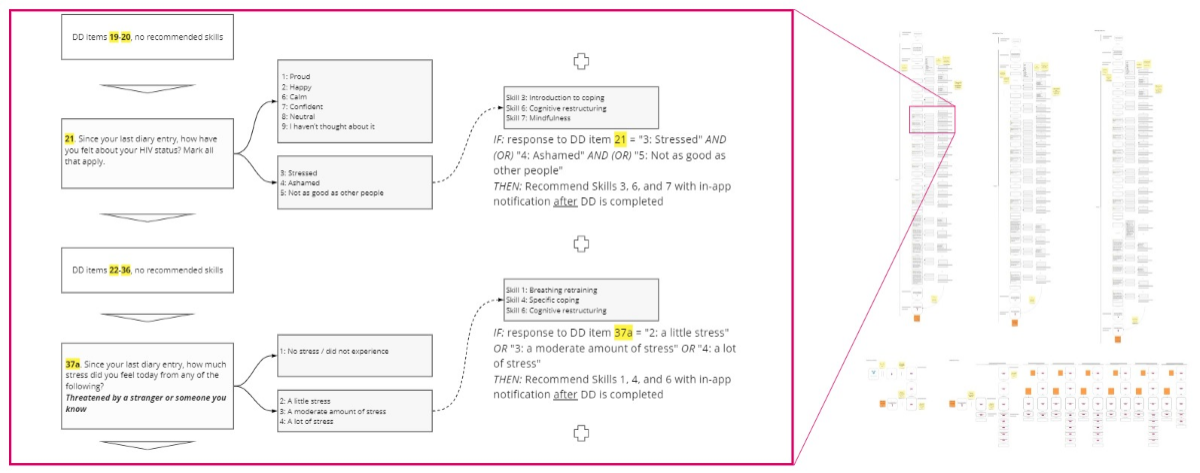
Selected user story map illustrating just-in-time adaptive intervention logic. DD: daily diary.

Subsequently, these JITAI logics were refined into user story maps, a user experience research technique that uses simple but summative flow diagrams to portray complex in-app interactions and contingencies [88]. An illustrative excerpt of the user story maps that we provided to our developers, Crowdbotics, is shown in Figure 2 (hosted in full resolution [106]).

The complete user story maps, encompassing all possible daily diary–coping skills recommendation–SkillCheck sequences, are provided in Multimedia Appendix 2 (which can be downloaded in full resolution [107]).

#### NOLA GEM App

Fundamental to a JITAI adaptation is the determination of (1) tailoring variables; (2) the timely provision of appropriate intervention content, mapped to a plausible contender proximal outcome, at each predefined threshold of a tailoring variable; and (3) clear, succinct, programmable logics defining the mechanisms of (1) and (2) [60,61]. NOLA GEM incorporates a subset of aim-1 daily diary inputs as tailoring variables. On the basis of aim-1 findings, the constructs and environmental stressors that will initialize the NOLA GEM app’s JITAI logics are mental health challenges; financial problems; *direct* violence experienced firsthand, such as an assault (with a built-in emergency response management protocol, if warranted); *indirect* violence, witnessed or overheard; *past* violence, resurfaced in the form of intrusive thoughts, memories, or trauma cognitions [82]; interpersonal discord or lack of social support; HIV-related stigma; alcohol-related craving and coping; and a general stress measure, the triggering of which will offer the end user a selection of all LIFT skills.

These stressors are operationalized via the daily diary measures piloted initially in aim 1. If the end user indicates an acute stressor during a daily diary beyond a prespecified cutoff value, they receive an in-app notification after completion of the daily diary inviting them to open a recommended LIFT skill. Should multiple postthreshold stressors be endorsed, the user is given the option to select their preferred LIFT skill. The microcopy of these messages is uniformly strengths-based, encouraging, and nonpathologizing (eg, “It looks like you might be dealing with some stress today. Based on your daily diary responses, NOLA GEM recommends reviewing these skills”). Embedded hyperlinks will automatically launch the recommended LIFT session if a user chooses to. Aiming to minimize “interaction cost,” or the cognitive load associated with navigating a user interface [108,109], multifaceted interactivity was largely eschewed in favor of brief, “snackable” content [110] in which practical skills are foregrounded, available in text and audio formats. Each session encapsulates the CTSC-based strategies taught by the manualized LIFT intervention [33,35]. NOLA GEM users are not required to complete each skill within a single session. Upon completion at a user-directed pace, end users are presented with single-item “SkillChecks,” which assess the degree to which each LIFT skillset was successfully imparted by the app and operationalize the proximal outcome to which the LIFT session and tailoring variable were mapped on the feature hypothesis chart using dedicated items in a Likert-type format. Structuring SkillChecks in this fashion further reduces interaction costs and permits both knowledge delivery and proximal outcomes to be examined as potentially distinct mediators of distal outcomes in aim 3.

In addition, supplanting the originally envisioned generalized geospatial JITAI triggers [79], NOLA GEM users, during onboarding to the app, can leverage a geofencing feature to prespecify areas or locations that reliably evince stress. With such geofencing established, entering the prespecified area will activate an SMS text message prompt (“It looks like you may be in a stressful place”) recommending each user’s favored LIFT coping skills. In addition, foregrounding resilience, an option to prespecify a place that end users associate with personal restorativeness is included.

**Figure 3:**
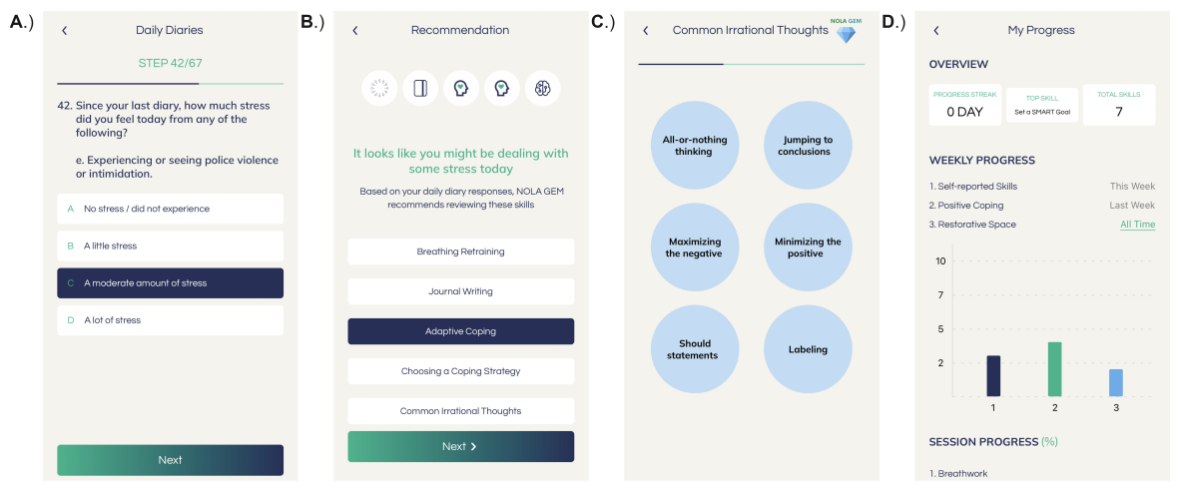
Illustrative screenshots of the NOLA GEM user interface. (A) sample daily diary item, (B) sample just-in-time adaptive intervention prompt, (C) Living in the Face of Trauma skill content, (D) “My Progress” tracker.

Illustrative screenshots of the NOLA GEM user interface are provided in Figure 3 (hosted in full resolution [111]).

### Aim 3: Acceptability, Feasibility, and Preliminary Efficacy

#### Trial Design

The pilot trial will be a single-blind parallel superiority trial assessing NOLA GEM (treatment arm) against GEMA alone (control arm) for a period of 21 days, allocated at a 1:1 ratio. In contrast to aim 1, the NOLA GEM app is capable of delivering SMS text message–linked daily diaries and recording GPS coordinates without recourse to third-party software. The GEMA-alone control was chosen to control for any intervention effects of recording daily mood, experiences, and other psychosocial variables as some research has demonstrated improvements in outcomes such as PTSD, depressive symptoms, and emotional awareness in participants recording their experiences using EMA methods [112-115] (aim-2 interviewees, unprompted, disclosed similar enhancements in self-awareness during their experience as aim-1 participants). This design establishes a high-resolution comparator to NOLA GEM, capturing GEMA data across both trial arms while potentially isolating the effects of JITAI-based momentary provision of LIFT content as a hypothesized mechanism of action.

#### Screening

The aim-3 inclusion criteria are equivalent to those of aims 1 and 2, although both HIV outpatient clinic–based as well as community-based recruitment strategies will be used, inviting people living with HIV across the New Orleans metropolitan area to learn about the study through flyers and screening in real time via a QR code. Once the code is scanned, users will be brought to a Qualtrics survey that will ask basic demographic questions to facilitate quota sampling, history of living with HIV, current ART medications, and past violence exposure, which will be assessed via the *Adverse Childhood Experiences Questionnaire* and US Department of Veterans Affairs National Center for PTSD *Life Events Checklist for DSM-5* [116].

An option to screen by phone, with a research assistant manually inputting each potential participant’s screener responses, will also be available. Once individuals are determined to meet the eligibility criteria, they will be called to set up an intake appointment.

#### Sample Size Considerations

The target sample size is approximately 100, which will accommodate an anticipated ≥10% attrition rate based on previous experience enrolling local violence-affected in-care people living with HIV, for an optimal analytic sample of 60 (30 per arm).

#### Study Settings

Participants will be recruited using flyers from several clinics and community locations in New Orleans that offer services to people living with HIV. Many of the community sites provide linkage to care by guiding people through insurance eligibility, appointments, and medication access. Two of these community sites were founded to provide services specifically to Black and African American women living with HIV. The clinic sites are federally qualified health centers that provide a range of care; however, we are working specifically with their HIV prevention and care teams.

By and large, aim 3 will comprise a digital trial subsequent to NOLA GEM onboarding. Screeners will be completed either on the participants’ phones or by calling research staff. Once participants are screened, intake appointments will take place either over Zoom or at private clinical facilities within the Tulane University School of Public Health and Tropical Medicine. All further aspects of the study will take place on the NOLA GEM app or over the phone.

#### Participants and Procedures

With eligibility determined, participants will be randomized, and a baseline appointment will be scheduled. Quota sampling will ensure a ≥50% female, 50% aged >40 years and 50% aged <40 years, and ≥75% Black sample, reflecting the unique drivers and PTSD symptom constellations endured by women and Black Americans while also reflecting New Orleans people living with HIV demographics.

At baseline, informed consent will be obtained, and an in-person or Zoom assessment will be administered by the research staff. The NOLA GEM app (through which participants can be assigned to either treatment or control user types via an integrated back end) will be installed on participants’ smartphones from the Crowdbotics-hosted Apple App Store or Google Play Store, and a detailed onboarding will be conducted by research staff to ensure uniform comprehension of the app’s features, the nature of GEMA, and the wording of daily diary items. Although participants will be instructed on the expectations, functional affordances, and user expectations specific to their trial arm, they will be blinded to their explicit treatment versus control assignment.

#### Smartphones

An iPhone SE smartphone will be provided to participants without access to a personal smartphone. Devices will be provided to participants with a scheduled return date. Once returned, iPhones will be sanitized and recharged, and all device histories (calls, browser, and SMS text message) will be cleared. If participants do not return the study-provided iPhone and become unreachable, they will be considered dropped from the study.

#### Data Collection and Management

The study app is Health Insurance Portability and Accountability Act (HIPAA) compliant and will transmit daily diary and GPS data to a Crowdbotics-hosted administrator panel, also HIPAA compliant, eschewing sensitive, identifiable data storage on participants’ personal devices. GPS data collection will occur continuously, recording latitudinal and longitudinal points at 1-minute intervals. In both arms, participants will be prompted twice each day by SMS text message to complete their daily diary items within a 3-hour window (9 AM-noon and 6 PM-9 PM) whenever ready and able to find privacy.

The aim-3 daily diary items are shown in Multimedia Appendix 3.

Throughout the conduct of the aim-3 trial, data will be securely stored on REDCap, and data sets that are downloaded for analysis will be stored on secure Tulane-hosted Box cloud servers. NOLA GEM user-level paradata, including daily diary responses, will be downloaded from the HIPAA-compliant Crowdbotics-hosted administrator panel, manually cleaned, and transformed into a standard “wide” data format in which each row corresponds to a unique participant using dedicated R scripts (R Foundation for Statistical Computing).

#### Data Safety Monitoring

All data collected will be monitored weekly by the research staff to ensure the privacy and confidentiality of the participants. General quality assurance checks will also be run on the data during and after collection. Any adverse events or unanticipated problems will be reported immediately to the institutional review board. To ensure the confidentiality of the data, each participant is assigned a unique identifier that will not be linked to any identifying information. Any technology used (Zoom, Qualtrics, NOLA GEM app, or REDCap) is HIPAA secure. All SMS text message–delivered reminders sent to participants during the research period will be generic and will have no specific content in case participants wish to keep their participation private.

#### Intervention Arm

Intervention arm participants will be provided access to the SMS text message prompts; daily diary interface; a resource list that covers local health care, housing, domestic violence, family, and mental health services; a “My Progress” screen where they can track elements of their interaction with the app; and the full suite of LIFT sessions, skills, and SkillChecks. Following the submission of each daily diary, the study app will recommend specific LIFT content based on participants’ particular patterns of momentary stressors. In addition, participants can access all content in their own time.

#### Control Arm (GEMA Alone)

Control arm participants will have access to the SMS text message and daily diary functions exclusively plus the resource list provided to treatment arm participants.

### Primary Outcomes

Primary, distal outcome measures will be assessed at the immediate postintervention time point (within 1 week of completing the 21-day intervention period) and the 30- and 90-day follow-up assessments, as specified in the following sections.

#### Acceptability, Feasibility, and Usability

JITAI-specific proximal outcomes have been summarized previously. The aim-3 distal outcomes and their precise operationalization are outlined in this section.

Feasibility and acceptability will be assessed after participation in the 21-day trial period. Feasibility will be operationalized via screening and enrollment rates; completion rates of daily diaries; and paradata describing within-person patterns of use, including JITAI-triggered prompts [61,117]. Acceptability will be operationalized as follows. Intervention arm participants will complete the *User Experience Questionnaire* (short version), with ratings of the NOLA GEM app’s pragmatic and hedonic quality User Experience Questionnaire domains assessed against available benchmarks [118]. The single-item *Net Promoter Score* will ask about participants’ likelihood to recommend NOLA GEM on a 0 to 10 scale [119]. Both measures will be administered at the immediate postintervention assessment.

Attrition rates will also be recorded, and those who drop out of the study will receive a set of questions exploring motivations for discontinuation wherever possible.

#### CoC Milestones

Primary outcomes, representing recognized HIV CoC milestones [1,21,23], will be operationalized as follows via a postassessment survey.

##### Care Engagement

Past-30-day outpatient retention will be assessed using the 12-item *HIV Treatment Adherence Self-Efficacy Scale*. A well-validated measure, the HIV Treatment Adherence Self-Efficacy Scale recognizes care engagement as a holistic, psychosocially, and environmentally contingent construct, with particular attention to personal routine [120]. In addition, participants will self-report the percentage of appointments they missed in the past 90 days at their 90-day follow-up assessment.

##### ART Adherence

The capacity to maintain ART regimens will be assessed using a novel single-item visual analog scale (VAS) [72]. The VAS asks participants to estimate the percentage of their medication that they have taken in the past 30 days. Details of the VAS format and development are provided by Giordano et al [121]. In addition, daily diary measures operationalizing use of and feelings toward ART medication will be administered over the 21-day trial period (eg, “Did you have difficulty fitting your HIV treatment into your daily routine yesterday [Yes/No]”). These measures are provided in full in Multimedia Appendix 3.

##### Viral Suppression

Virologic control will be assessed via a self-report item asking participants whether their most recent HIV viral load test result was <50 copies/mL at the 90-day follow-up assessment. This item is adapted from the study by Carter et al [122].

### Secondary Outcomes

#### Mental Well-Being

Diverse domains of psychosocial and emotional well-being will be assessed among all aim-3 participants.

##### PTSD Assessment

At 30 days after baseline, the *PTSD Checklist for DSM-5*, a 20-item self-report measure that assesses the presence and acuity of *DSM-5* PTSD symptoms, will assess traumatic stress burden for participants [123]. Considered an acceptable means of provisional diagnosis, the PTSD Checklist for DSM-5 has demonstrated psychometric soundness and acceptable continuity between Diagnostic and Statistical Manual of Mental Disorders, Fourth Edition, and *DSM-5* PTSD conceptualizations [124]. In addition, a range of daily diary measures (detailed in Multimedia Appendix 3) will record daily fluctuations in posttraumatic stress symptom acuity.

##### Anxiety and Depression

At 30 days after baseline, the 14-item *Hospital Anxiety and Depression Scale* will assess the presence and severity of anxiety and depression symptoms. Fielded and assessed for decades [125], the Hospital Anxiety and Depression Scale is shown to possess acceptable construct validity and internal consistency among clinical samples [126]. In addition, a mood checklist is administered via the daily diaries prompting participants to mark all moods they have experienced in the interim since their last daily diary (eg, “excited,” “relaxed,” or “scared”; Multimedia Appendix 3).

##### Effective Coping

At 30 days after baseline, the Brief *Coping Orientation to Problems Experienced* and *Coping Self-Efficacy Scale* will measure participants’ recourse to adaptive versus maladaptive styles of coping [127]. Translated into French, Spanish, Malay, and Russian and fielded successfully among broadly heterogeneous populations, the Brief Coping Orientation to Problems Experienced consistently evidences acceptable psychometric properties, although its internal factor structure remains contested [128]. The Coping Self-Efficacy Scale also demonstrates good reliability and validity, including among samples of in-care people living with HIV [129]. Via a checklist, coping strategies (“getting emotional support from others” and “criticizing myself”) will be logged in the aim-3 daily diaries (Multimedia Appendix 3).

##### Hazardous Substance Use

At 30 days after baseline, the 3-item *Alcohol Use Disorders Identification Test*, a valid instrument for detecting heavy, potentially unhealthy alcohol use patterns [130], will be applied to that end in aim 3. The “Alcohol/Drugs” section of the widely used *Addiction Severity Index* will assess the effects of participants’ alcohol and drug use across key domains of daily functioning [131]. Dedicated daily diary items will also prompt participants to describe patterns of alcohol and drug use since “last night” (morning daily diary) or “your last daily diary” (afternoon daily diary; Multimedia Appendix 3).

##### Domain–General Stress

Finally, at 30 days after baseline, the *Perceived Stress Scale–4* will measure situational subjective stress among aim-3 participants [132]. In addition, a 24-item aim 1–generated novel daily stressor severity checklist operationalizing a wide array of potential sources of stress arousal (eg, community and police violence, intrafamilial strife, and employment challenges) will assess multidomain stress experienced by aim-3 participants at the 30-day follow-up relative to their baseline stress burden.

#### Analytic Approach

Consistent with aim 1, aim-3 data will be cleaned and saved in SAS data files (SAS Institute), with extensive consistency checks conducted at all levels of data [66]. At the completion of each check, the data will be added to the existing database for analysis. GPS data will be stored on a password-protected server and constructed into activity paths, replicating the aim-1 approach [66].

Preliminarily, descriptive and crude bivariate associations between variables of interest will be generated. Daily diary completion rates and agreement with aggregate retrospective survey data for comparable measures will be calculated. Data reduction techniques, mitigating the high volume and dimensionality of GEMA-derived data, will include the creation of composite indexes of cumulative risk, with exploratory factor analysis, assessment of internal consistency, and Bonferroni correction applied as warranted.

Aim-3 analyses of preliminary efficacy will include analysis of each day-to-day distal outcome as a continuous variable as well as average exposures across the total intervention period. We will explore potential transformations to approach normality on key response variables [133]. Linear mixed-effects models will relate day-level outcomes to day-level indexes of cumulative risk and protective exposure derived originally from aim 1, adjusted for baseline values [134]. Random effects will be included to account for within-subject series correlations. Control variables will be modeled as fixed effects.

### Ethics Approval

All methods and materials were reviewed and approved by the Biomedical and Social Behavioral Institutional Review Boards of Tulane University (aim 1: 860463; aim 2: 2021-1220; and aim 3: 2022-1861).

## Results

Aim 1, enrolling 89 people living with HIV, was completed in September 2022. Aim 2, informed by a pilot aim-1 sample of 49 participants and a subset of 12 interviewees, was completed with the handover of the NOLA GEM app to investigators in April 2022. NOLA GEM is available to aim-3 participants as a native iOS and Android app hosted in the Apple App Store and Google Play Store, respectively. The app’s onboarding and sign-on flow accommodates both intervention (treatment arm) and GEMA-only (control arm) account credentials. Ethics approval was obtained, and recruitment subsequently began, in July 2023. We anticipate that all follow-up appointments will be completed by February 2024, with the final results ready for dissemination by May 2024. The results will be disseminated to key stakeholders via scientific conferences, peer-reviewed academic journals, and Tulane University social media channels. Ultimately, as an R34-supported project, the longer-term objective is to “prune” underused or preliminarily inefficacious JITAI logics to refine a parsimonious NOLA GEM theory of change, consequently supporting an application for National Institutes of Health support for a full-scale RCT.

## Discussion

### Summary

This study aims to advance knowledge on temporally fluctuant and place-based stressors endured by people living with HIV in New Orleans and assess the acceptability, feasibility, and preliminary efficacy of NOLA GEM, a JITAI-consistent mHealth intervention responsive to these stressors. Key to the significance of NOLA GEM is its capacity to capture and intervene in real time on a holistic range of time-variant psychosocial and behavioral variables [10,15,21,60,61] alongside crucial neighborhood-level factors known to affect CoC outcomes and well-being among people living with HIV [11,12,23,44-48], particularly violence-affected people living with HIV [1,4,7,15]. To our knowledge, NOLA GEM is the first smartphone-delivered, trauma-informed intervention tailored for people living with HIV. As such, it will leverage a well-demonstrated EBI [33,34,36-39]; previous research supporting cognitive behavioral touch points for intervention among people living with HIV with trauma histories [135-137]; and the confidentiality, discretion, and real-time accessibility of mHealth [54,86]. Through the latter strengths in particular, we hope to address participant privacy and in-person group attendance concerns that may have stemmed the uptake and, possibly by extension, the longer-term efficacy of *Improving AIDS Care after Trauma* [39], the LIFT adaptation preceding NOLA GEM [38].

Notably, the mean ages of aim-1 and aim-2 participants were 57 and 58 years, respectively. This skew toward older adulthood relative to the intended user base of many people living with HIV–tailored mHealth interventions [138] may have influenced the design of NOLA GEM. Since 2014, smartphone adoption has accelerated among older adults, reaching 86% among adults aged >50 years and 62% among adults aged >70 years [139]. With motivation (both intrinsic and extrinsic) and proper feedback and support, mHealth and eHealth tools can find success among older adults [140], including older people living with HIV who, for example, engaged with the features of the *PositiveLinks* platform in a significantly more dedicated, sustained manner than younger (aged <50 years) users [141]. Success among this user base relies on a baseline degree of digital readiness [140], which is itself often contingent on device familiarity—a strength of NOLA GEM, which leverages common SMS text message and native app functional affordances, distinct from, for example, a wearable actigraphy sensor. Though some aim-2 participants attached their preferred coping techniques to their age (accepting and adapting: “You’re getting older and you can’t do anything about it. Just accept it and let move on” [participant F]), few, when prompted, described personal traits such as age or disability influencing their personal technology use. Post hoc, open-ended usability interviewing conducted during or shortly after intervention offboarding will allow us to examine the role of participant age (among both younger and older adults) qualitatively within the specific context of the NOLA GEM user experience.

Overarchingly, our objective is to introduce an innovative model of adaptive coping support in a periurban US Gulf South context characterized by elevated rates of HIV prevalence [41], poverty and economic inequality [42,43], and violent crime [44], reinforcing a focus on traditional HIV CoC end points with precision-tailored support for person-centered proximal outcomes grounded in the lived experience of local people living with HIV.

### Limitations

As a small-scale pilot trial, aim 3 is subject to a number of inherent limitations. First, and by design [142], it is underpowered to examine efficacy in a formal hypothesis-driven manner. Mediation analyses of proximal outcomes will necessarily be exploratory in nature and inform the causal pathway hypotheses for the planned RCT. Although the selection of control conditions for mHealth trials remains an area of active investigation and debate [143], a passive GEMA-alone control may inflate preliminary estimates of NOLA GEM (incorporating GEMA+LIFT coping skills) efficacy on primary outcomes, an issue that is potentially exacerbated by the small sample size [144]. Adaptations of complex, sequential, and multicomponent group interventions risk dismantling key mechanisms of action that undergird effectiveness in their original contexts [33,34,101]. Adaptation to mHealth across HIV science remains a highly active but often methodologically impromptu area [145]. Pragmatic considerations required us to deprecate certain components of LIFT as originally manualized [33] and demonstrated to reduce traumatic stress [34,36]. Group cohesion and dedicated in-person facilitation of the group member disclosure and support processes define the LIFT intervention trajectory, which is sequenced across 15 sessions [33]. We abstracted the CTSC-based strategies built into NOLA GEM from their original, in-person, session-by-session framework to activate the “just-in-time” aspect of a JITAI adaptation while mitigating ethical concerns that, if faced with an acute stressor, a NOLA GEM user would be denied the best-suited coping support if they had not yet reached the appropriate session had a strictly linear intervention model been retained. Similarly, although a peer-to-peer support forum or social media–style feature [146,147] in which users could interact pseudonymously was briefly considered, lacking active human (vs algorithmic) content moderation, such a space could become consumed with off-topic, inane, or even outrage-inducing user-generated content and external media [86,148]. In addition, by recruiting people living with HIV who were either violence-affected individually or lived in a high-violence neighborhood, a degree of heterogeneity in needs was introduced into the aim-1 and aim-2 samples, which may have swayed the formative insights—and, by extension, intervention design—derived from the *New Orleans Alcohol Use in HIV* study subsamples. Although violence exposure at the within-person and neighborhood levels is associated with distinct sequelae among people living with HIV, it is our intention that the multitude of possible NOLA GEM user journeys can offer the necessary support to a wide range of personal violence histories. By nature, an acceptability and feasibility trial will provide insights on many of these concerns, whereas merged paradata and GEMA data will permit high-resolution analyses of users’ engagement with and navigation of NOLA GEM [149].

### Conclusions

Aligning user-centered development practices with principles that elevate the lived expertise of people living with HIV, mHealth-adapted EBIs can sustain well-specified theories of change while leveraging the affordances of mobile technologies. The results of the aim-3 trial will generate needed evidence concerning the value of such a locally tailored JITAI approach while pointing toward opportunities to improve upon the initial release of NOLA GEM in anticipation of a fully powered RCT offering definitive evidence of the potential of mHealth-delivered effective coping support that operationalizes the self-knowledge of communities of end users.

## Appendix

Multimedia Appendix 1 Aim-1 daily diary measures—domains and operationalization.

Multimedia Appendix 2 Complete NOLA GEM just-in-time adaptive intervention user story maps.

Multimedia Appendix 3 Aim-3 daily diary measures—domains and operationalization.

## Abbreviations

ART: antiretroviral therapy
CoC: continuum of care
CTSC: cognitive theory of stress and coping
DSM-5: Diagnostic and Statistical Manual of Mental Disorders, Fifth Edition
EBI: evidence-based intervention
EMA: ecological momentary assessment
GEMA: geographic ecological momentary assessment
HIPAA: Health Insurance Portability and Accountability Act
JITAI: just-in-time adaptive intervention
LIFT: Living in the Face of Trauma
mHealth: mobile health
PTSD: posttraumatic stress disorder
UX: user experience
RCT: randomized controlled trial
REDCap: Research Electronic Data Capture
SPIRIT: Standard Protocol Items: Recommendation for Interventional Trials
SUD: substance use disorder
VAS: visual analog scale

## Data Availability

Deidentified aim-1 data are deposited and deidentified aim-3 data will be deposited upon completion of the pilot trial on the National Institute on Alcohol Abuse and Alcoholism Data Archive of the National Institute of Mental Health Data Archive [150].
