# Supplementary material for: A Trauma-Informed, Geospatially Aware, Just-in-Time Adaptive mHealth Intervention to Support Effective Coping Skills Among People Living With HIV in New Orleans: Development and Protocol for a Pilot Randomized Controlled Trial
Source: JMIR Res Protoc. 2023 Oct 24;12:e47151. doi: 10.2196/47151 (PMC10630874; doi:10.2196/47151)
Supplement: Multimedia Appendix 1 [file resprot_v12i1e47151_app1.pdf]

AIM I: 8AM MORNING DAILY DIARY

Type of Q	Type Q #	Logic	Question	Response Type	Response Options
Overall Location	1		How would you describe your current location overall?	Multiple choice	"workplace", "inside your home", "inside another's home", "vehicle/bus", "outside", "other" (write-in option if "other" chosen)
Overall Location	2		Who are you with currently?	Mark all that apply	"no one", "family member", "friends", "significant other", "strangers", "acquaintances", "coworkers"
Overall Location	3		How many plants, trees or parks do you currently see in your location ?	1-3 range	1=none 2=some 3= a lot
Overall Location	4		How much do you see people talking, socializing, or interacting currently in your location?	1-3 range	1=none 2=some 3= a lot
Overall Location	5		How much litter/trash on the streets or sidewalks do you currently see in your location?	1-3 range	1=none 2=Some 3=a lot
Overall Location	6		How much drug use /alcohol use and/or drug dealing do you currently see in your location?	1-3 range	1=none 2=some 3= a lot

Overall Location	7		How safe do you feel in your current environment?	1-3 range	1=not at all safe, 2=somewhat safe, 3=very safe
Safety Trigger					
Overall Location	8		How easy would it be for you to get alcohol if you wanted some where you are right now?	1-3 range	1=not easy at all, 2=somewhat easy, 3=very easy
Overall Location	9		How easy would it be for you to get any drugs/illegal substances if you wanted some where you are right now?	1-3 range	1=not at all, 2=somewhat, 3=very
Overall Location	10		Where did you go yesterday?	Check all that apply.	_1=grocery store or other store<br>_2=work<br>_3=friend or family member's house<br>_4=social outing<br>_5=restaurant, bar, or club<br>_6=exercise or recreation<br>_7=other<br>_8=didn't leave house
Mood Stress	1		Since your last diary entry/last night, how have you felt?	Mark all that apply	"excited", "angry", "happy", "sad", "stressed", "tired", "relaxed", "bored", "irritated", "proud", "strong", scared, guilty, grateful, drunk/high, in pain, craved alcohol, craved other non-prescription drug/substance, hungover, ill/sick "none of the above"
Mood Stress	2		To what degree are your emotions	1-3 range	1=not out of control, 2=somewhat out of control 3= very out of control

			feeling out of control right now?		
Mood Stress	3		How much stress are you feeling right now?	1-3 range	1=no stress 2=some stress 3=a lot of stress
Stigma	1		Since last night/your last diary entry, have you felt like people treated you unfairly?	Binary	Yes/No
Stigma	2	If Yes to Stigma 1	Do you feel that this due to any of the following [check all that apply]?	Mark all that apply	“Your sex or gender”, “your sexual orientation”, “your race or ethnicity”, “your alcohol or drug use”, “your HIV status” "other"
Stigma	3		Since last night/your last diary entry, how have you felt about your HIV status?	Mark all that apply	Proud, Happy, Stressed, Ashamed, Not as good as other people, Calm, Confident, Neutral, I haven't thought about it
Substance Use	1		Since last night/your last diary entry, how many drinks containing alcohol have you had?	# of drinks	None<br>1 -2<br>3 -4<br>5-7<br>8+
Substance Use	2		Since last night/your last diary entry, how many times have you used drugs/substances other than alcohol	# of times	None<br>1 -2<br>3 -4<br>5-7<br>8+

			such as marijuana, cocaine, etc.?		
Substance Use	3	If Substance Use 1 or 2 =/0, then show	Who did you drink or get high with?	Mark all that apply	“no one”, “family member”, “friends”, “significant other”, “strangers”, “acquaintances”
Substance Use AM ONLY	1		Please select the substances that you used yesterday.	mark all that apply	Alcohol; Cannabis/marijuana; Cocaine/crack; Opiates (heroin, methadone, oxycontin, fentanyl); Other: please specify; No substances
Substance Use AM ONLY	2	If anything selected for SU AM ONLY 1	How intoxicated were you yesterday at your peak while using alcohol or any other substance?	1-3 range	1=not at all, 2=some 3=very
Prompt		If anything selected for SU AM ONLY 1	You reported that you drank alcohol or took a drug yesterday. Did you drink or take drugs because:	Mark all that apply.	
Drinking Motives	1	If anything selected for SU AM ONLY 1	My friends or family pressured me to do it	checklist	
Drinking Motives	2	If anything selected for SU AM ONLY 1	It helped me when I felt depressed or nervous	checklist	
Drinking Motives	3	If anything selected for SU AM ONLY 1	It cheered me up when I was in a bad mood	checklist	
Drinking Motives	4	If anything selected for SU AM ONLY 1	It gave me a pleasant feeling	checklist	

Drinking Motives	5	If anything selected for SU AM ONLY 1	It improved a party or celebration	checklist	
Drinking Motives	6	If anything selected for SU AM ONLY 1	It helped me forget about my problems	checklist	
Drinking Motives	7	If anything selected for SU AM ONLY 1	It helped me forget about my health problems	checklist	
Drinking Motives	8	If anything selected for SU AM ONLY 1	Other, please specify:	checklist	
Drinking Motives	9	If anything selected for SU AM ONLY 1	None of the above	checklist	
HIV Specific	1		Did you take your prescribed HIV medication(s) yesterday?	1-3	1=None of my HIV medication<br>2=Some of y HIV medication<br>3=All of my HIV medication
HIV Specific	2		Did you have difficulty fitting your HIV treatment into your daily routine yesterday?	Binary	Yes/No
HIV Specific	3		Were you tempted NOT to take your ART medication yesterday because you were drunk or high?	Binary	Yes/No
HIV Specific	4		Were you tempted NOT to take your ART medication yesterday because	Binary	Yes/No

			you were not feeling well?		
HIV Specific	5		Did your friends encourage you to take your ART meds yesterday?	Binary	Yes/No
HIV Specific	6		Did a partner/family member encourage you to take your ART meds yesterday?	Binary	Yes/No
Prompt			**Following are some feelings people might have when managing their HIV medications daily. Please let us know if you were feeling that way yesterday.**		
HIV Specific	5		It frustrated me to think that I will have to take these HIV medications every day for the rest of my life.	Binary	Yes/No
HIV Specific	6		I felt in control of this disease by taking my HIV medications as prescribed	Binary	Yes/No
Mini-Stress	1		Since last night/your last diary entry, have	Binary	Yes/No

			you had a stressful experience?		
Mini-Stress	2	Skip if no to mini-stress 1	How much do you believe you can cope with your most stressful experience since last night/your last diary entry?	1-3 range	1=not at all, 2=somewhat, 3=very much
Daily Appraisals	1	Skip if no to mini-stress 1	How stressful was this event?	1-3 range	1=not very stressful, 2=somewhat stressful, 3=very stressful
Daily Appraisals	2	Skip if no to mini-stress 1	How much do you feel you can control the outcome of this event?	1-3 range	1=not at all, 2=somewhat, 3=very much (doesn't apply)
Sleep Quality	1		How satisfied were you with your sleep last night?	1-3	1= not at all satisfied, 2=somewhat satisfied, 3= very satisfied

AIM I: 1PM DAILY DIARY

Type of Q	Type Q #	Logic	Question	Response Type	Response Options
Overall Location	1		How would you describe your current location overall?	Multiple choice	"workplace", "inside your home", "inside another's home", "vehicle/bus", "outside", "other" (write-in option if "other" chosen)
Overall Location	2		Who are you with currently?	Mark all that apply	"no one", "family member", "friends", "significant other", "strangers", "acquaintances", "coworkers"
Overall Location	3		How many plants, trees or parks do	1-3 range	1=none 2=some 3= a lot

			you currently see in your location ?		
Overall Location	4		How much do you see people talking, socializing, or interacting currently in your location?	1-3 range	1=none 2=some 3= a lot
Overall Location	5		How much litter/trash on the streets or sidewalks do you currently see in your location?	1-3 range	1=none 2=Some 3=a lot
Overall Location	6		How much drug use /alcohol use and/or drug dealing do you currently see in your location?	1-3 range	1=none 2=some 3= a lot
Overall Location	7		How safe do you feel in your current environment?	1-3 range	1=not at all safe, 2=somewhat safe, 3=very safe
Safety Trigger					
Overall Location	8		How easy would it be for you to get alcohol if you wanted some where you are right now?	1-3 range	1=not easy at all, 2=somewhat easy, 3=very easy
Overall Location	9		How easy would it be for you to get any drugs/illegal substances if you wanted some	1-3 range	1=not easy at all, 2=somewhat easy, 3=very easy

			where you are right now?		
Mood Stress	1		Since your last diary entry, how have you felt?	Mark all that apply	"excited", "angry", "happy", "sad", "stressed", "tired", "relaxed", "bored", "irritated", "proud", "strong", scared, guilty, grateful, drunk/high, in pain, craved alcohol, craved other non-prescription drug/substance, hungover, ill/sick "none of the above"
Mood Stress	2		To what degree are your emotions feeling out of control right now?	1-3 range	1=not out of control, 2=somewhat out of control 3= very out of control
Mood Stress	3		How much stress are you feeling right now?	1-3 range	1=no stress 2=some stress 3=a lot of stress
Stigma	1		Since your last diary entry, have you felt like people treated you unfairly?	Binary	Yes/No
Stigma	2	If Yes to Stigma 1	Do you feel that this due to any of the following [check all that apply]?	Mark all that apply	"Your sex or gender", "your sexual orientation", "your race or ethnicity", "your alcohol or drug use", "your HIV status" "other"
Stigma	3		Since your last diary entry, how have you felt about your HIV status?	Mark all that apply	Proud, Happy, Stressed, Ashamed, Not as good as other people, Calm, Confident, Neutral, I haven't thought about it
Substance Use	1		Since /your last diary entry, how many drinks containing alcohol have you had?	# of drinks	None<br>1 -2<br>3 -4<br>5-7<br>8+

Substance Use	2		Since your last diary entry, how many times have you used drugs/substances other than alcohol such as marijuana, cocaine, etc.?	# of times	None<br>1 -2<br>3 -4<br>5-7<br>8+
Substance Use	3	If Substance Use 1 or 2 =/0, then show	Who did you drink or get high with?	Mark all that apply	“no one”, “family member”, “friends”, “significant other”, “strangers”, “acquaintances”
Intent	1		Do you intend to drink any drinks containing alcohol today?	Numeric	Yes, No, I have already drank today
Intent	2		Do you intend to use any drugs/substances such as marijuana, cocaine, etc. today?	Numeric	Yes, No, I have already used today
Mini-Stress	1		Since your last diary entry, have you had a stressful experience?	Binary	Yes/No
Mini-Stress	2	Skip if no to mini-stress 1	How much do you believe you can cope with your most stressful experience since your last diary entry?	1-3 range	Not at all, Somewhat, Very much
Daily Appraisals	1	Skip if no to mini-stress 1	How stressful was this event?	1-3 range	1=not very stressful, 2=somewhat stressful, 3=very stressful<br>(doesn’t apply)

Daily Appraisals	2	Skip if no to mini-stress 1	How much do you feel you can control the outcome of this event?	1-3 range	1=not at all, 2=somewhat, 3=very much (doesn't apply)

AIM I: 7PM DAILY DIARY

Type of Q	Type Q #	Logic	Question	Response Type	Response Options
Overall Location	1		How would you describe your current location overall?	Multiple choice	"workplace", "inside your home", "inside another's home", "vehicle/bus", "outside", "other" (write-in option if "other" chosen)
Overall Location	2		Who are you with currently?	Mark all that apply	"no one", "family member", "friends", "significant other", "strangers", "acquaintances", "coworkers"
Overall Location	3		How many plants, trees or parks do you currently see in your location ?	1-3 range	1=none 2=some 3= a lot
Overall Location	4		How much do you see people talking, socializing, or interacting currently in your location?	1-3 range	1=none 2=some 3= a lot
Overall Location	5		How much litter/trash on the streets or sidewalks do you currently see in your location?	1-3 range	1=none 2=Some 3=a lot
Overall Location	6		How much drug use /alcohol use and/or drug dealing do you currently see in your location?	1-3 range	1=none 2=some 3= a lot
Overall Location	7		How safe do you feel in your current environment?	1-3 range	1=not at all safe, 2=somewhat safe, 3=very safe
Safety Trigger					

Overall Location	8		How easy would it be for you to get alcohol if you wanted some where you are right now?	1-3 range	1=not easy at all, 2=somewhat easy, 3=very easy
Overall Location	9		How easy would it be for you to get any drugs/illegal substances if you wanted some where you are right now?	1-3 range	1=not easy at all, 2=somewhat easy, 3=very easy
Mood Stress	1		Since your last diary entry, how have you felt?	Mark all that apply	“excited”, “angry”, “happy”, “sad”, “stressed”, “tired”, “relaxed”, “bored”, “irritated”, “proud”, “strong”, scared, guilty, grateful, drunk/high, in pain, craved alcohol, craved other non-prescription drug/substance, hungover, ill/sick “none of the above”
Mood Stress	2		To what degree are your emotions feeling out of control right now?	1-3 range	1=not out of control, 2=somewhat out of control 3= very out of control
Mood Stress	3		How much stress are you feeling right now?	1-3 range	1=no stress 2=some stress 3=a lot of stress
Stigma	1		Since your last diary entry, have you felt like people treated you unfairly?	Binary	Yes/No
Stigma	2	If Yes to Stigma 1	Do you feel that this due to any of the following [check all that apply]?	Mark all that apply	“Your sex or gender”, “your sexual orientation”, “your race or ethnicity”, “your alcohol or drug use”, “your HIV status” "other"
Stigma	3		Since your last diary entry, how have you felt about your HIV status?	Mark all that apply	Proud, Happy, Stressed, Ashamed, Not as good as other people, Calm, Confident, Neutral, I haven't thought about it
Substance Use	1		Since your last diary entry, how many drinks containing alcohol have you had?	# of drinks	None<br>1 -2<br>3 -4<br>5-7<br>8+

Substance Use	2		Since your last diary entry, how many times have you used drugs/substances other than alcohol such as marijuana, cocaine, etc.?	# of times	None<br>1 -2<br>3 -4<br>5-7<br>8+
Substance Use	3	If Substance Use 1 or 2 =/0, then show	Who did you drink or get high with?	Mark all that apply	"no one", "family member", "friends", "significant other", "strangers", "acquaintances"
	1		The next few questions ask about difficult events you might have experienced today. Please mark all that you experienced today .:		
Stressor/Exposure			Threatened by a stranger or someone you know	checklist	
Stressor/Exposure			Hit, kicked, or physically injured or experienced unwanted sexual contact	checklist	
Stressor/Exposure			Saw someone get robbed, injured, or threatened.	checklist	
Stressor/Exposure			Heard gunfire.	checklist	
Stressor/Exposure			Experienced or saw police violence or intimidation.	checklist	
Stressor/Exposure			Had an argument with friend, family member, or romantic partner	(checklist)	
Stressor/Exposure			Dealt with an illness, injury, or accident	(checklist)	
Stressor/Exposure			Had problems with work	(checklist)	
Stressor/Exposure			Had a problem with money	(checklist)	

Stressor/Exposure			Didn’t feel supported by friends/family	(checklist)	
Stressor/Exposure			Experienced stress or chaos in my environment	(checklist)	
Stressor/Exposure			Dealt with COVID related stress	(checklist)	
Stressor/Exposure			Had a transportation problem	(checklist)	
Stressor/Exposure			Had to carefully watch what I say and how I say it	(checklist)	
Stressor/Exposure			Worried about running out of food	(checklist)	
Stressor/Exposure			Worried about paying rent or getting evicted	(checklist)	
Stressor/Exposure			Worried about my mental health	(checklist)	
Stressor/Exposure			Worried about my physical health	(checklist)	
Stressor/Exposure			Experienced problems/Issues with health care	(checklist)	
Stressor/Exposure			Had stress related to childcare or caregiving	(checklist)	
Stressor/Exposure			Other stressful or difficult event, please specify:	(checklist)/fill in	
Stressor/Exposure			No stressful or difficult events.	(checklist)	
Violence & Mental Health Trigger					
Stressor/Exposure	2	Carry forward checked from DS1	Which of these stressful experiences bothered you the most?	Multiple choice	Checked answers will move forward to select from
Daily Appraisals	1	If answered no stressors for stressor/exposure section, then skip	How much do you feel you can control the outcome of this event?	1-3 range	1=not at all, 2=somewhat, 3=very

Daily Appraisals	2	If answered no stressors for stressor/exposure section, then skip	How stressful was this event?	1-3 range	1=not at all stressful, 2=somewhat stressful, 3=very stressful
Prompt			Have you done any of the following today to deal with the most stressful experience you had today?		
Daily Coping	1		concentrating my efforts on doing something about the situation I'm in	(checklist)	
Daily Coping	2		getting emotional support from others	(checklist)	
Daily Coping	3		refusing to believe that it has happened	(checklist)	
Daily Coping	4		using alcohol or other drugs to help me get through it	(checklist)	
Daily Coping	5		criticizing myself	(checklist)	
Daily Coping	6		trying to come up with a strategy about what to do	(checklist)	
Daily Coping	7		looking for something good in what is happening	(checklist)	
Daily Coping	8		doing something to think about it less, such as going to movies, watching TV, reading, daydreaming, sleeping, or shopping	(checklist)	
Daily Coping	9		accepting the reality of the fact that it has happened	(checklist)	
Daily Coping	10		expressing my negative feelings	(checklist)	
Daily Coping	11		trying to find comfort in my religion or spiritual beliefs	(checklist)	

Daily Coping	12		trying to get advice or help from other people about what to do	(checklist)	
Daily Coping	13		blaming myself for things that happened	(checklist)	
Daily Coping	14		praying or meditating	(checklist)	
Daily Coping	15		Other (please specify)	(checklist)	
Prompt			Answer the following statements based on your experiences today.		
PTSD	1		How much were you bothered by disturbing thoughts, activities, or feelings about difficult life events that have happened to you?	1-5 range	1= not at all, 2=a little bit, 3=moderately, 4=quite a bit, 5=extremely
PTSD	2		How much were you bothered by avoiding thoughts, activities, or feelings about difficult life events that have happened to you?	1-5 range	1= not at all, 5=extremely
PTSD	3		How much were you bothered by feeling distant or cut off from other people and/or feeling emotionally numb?	1-5 range	1= not at all, 5=extremely
PTSD	4		How much were you bothered by difficulty concentrating, feeling jumpy or easily startled, feeling overtly alert, or feeling irritable or angry?	1-5 range	1= not at all, 5=extremely
PTSD	5		How much were you bothered by having strong negative beliefs about yourself, other people, or the world (for	1-5 range	1= not at all, 5=extremely

			example, having thoughts such as: I am bad, there is something seriously wrong with me, no one can be trusted, the world is completely dangerous)?		
Prompt			How much did you believe these statements to be true today?		
Post Trauma Cognitions	1		I am a good person	Binary	Yes/No
Post Trauma Cognitions	2		I don't trust anyone anymore	Binary	Yes/No
Post Trauma Cognitions	3		I trust my own judgement	Binary	Yes/No
Post Trauma Cognitions	4		I feel as though I can depend on other people	Binary	Yes/No
Post Trauma Cognitions	5		Most people are basically caring	Binary	Yes/No
Prompt			How often did you use the following strategies to deal with stress today?		
Emotion Regulation	1		I thought over and over again about my emotions	1-3 range	1= Not at all 2=Sometimes 3=A lot of the time
Emotion Regulation	3		I was careful not to express my emotions to others	1-3 range	1= Not at all 2=Sometimes 3=A lot of the time
Emotion Regulation	4		I accepted my emotions as valid and important	1-3 range	1= Not at all 2=Sometimes 3=A lot of the time
Emotion Regulation	5		I ignored my emotions	1-3 range	1= Not at all 2=Sometimes 3=A lot of the time

Physical Activity	1		What type of physical activity did you do today, if any?		1=None, 2= moderate physical activity or walking that increases your heart rate or makes you breathe harder than normal? (Ex. Bicycling at a regular pace, carrying light loads, mowing the lawn), 3= vigorous physical activity that makes you sweat or breathe/pant heavily? (Ex. Jogging, heavy lifting, fast bicycling, aerobics), 4=other
COVID	1		Did the novel coronavirus or covid-19 affect your ability to socialize today, such as seeing friends or going out?	Binary	Yes/No.
COVID	2		Did the novel coronavirus or covid-19 affect your movement, such as where you went or how you got around?	Binary	Yes/No.
Positive Social Interaction			How many social interactions (in person, over the phone, or electronically) did you have today that made you feel good or happy?	Text	
Motivation	1		What if anything motivated you today to take care of your health?	Text	Open response
