# Supplementary figures and images for: A Trauma-Informed, Geospatially Aware, Just-in-Time Adaptive mHealth Intervention to Support Effective Coping Skills Among People Living With HIV in New Orleans: Development and Protocol for a Pilot Randomized Controlled Trial
Source: JMIR Res Protoc. 2023 Oct 24;12:e47151. doi: 10.2196/47151 (PMC10630874; doi:10.2196/47151)

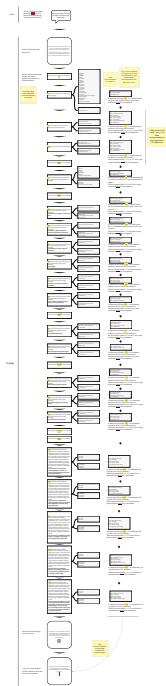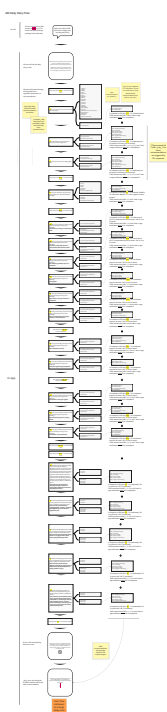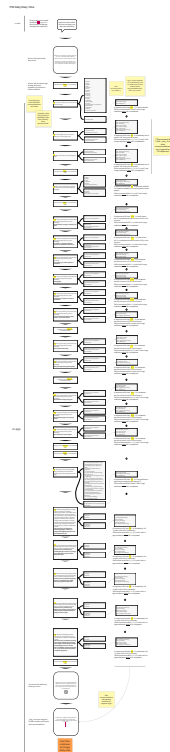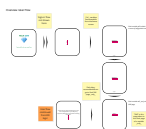

Control System

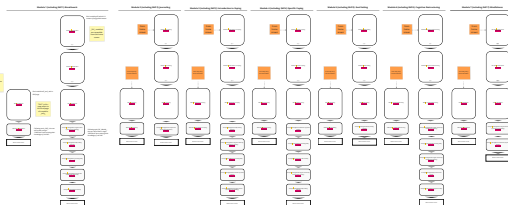

Supplement: Multimedia Appendix 2 [file resprot_v12i1e47151_app2.pdf]
