# Supplementary material for: A Trauma-Informed, Geospatially Aware, Just-in-Time Adaptive mHealth Intervention to Support Effective Coping Skills Among People Living With HIV in New Orleans: Development and Protocol for a Pilot Randomized Controlled Trial
Source: JMIR Res Protoc. 2023 Oct 24;12:e47151. doi: 10.2196/47151 (PMC10630874; doi:10.2196/47151)
Supplement: Multimedia Appendix 3 [file resprot_v12i1e47151_app3.pdf]

AIM III: 9AM MORNING DAILY DIARY

Type of Q	Type Q #	Logic	Question	Response Type	Response Options
Overall Location	1		How would you describe your current location overall?	Multiple choice	“workplace”, “inside your home”, “inside another’s home”, “vehicle/bus”, “outside”, “other (please specify)” (write-in option if “other” chosen)
Overall Location	2		Who are you with currently?	Mark all that apply	“no one”, “significant other”, “family member”, “friends”, “acquaintances”, “coworkers”, “strangers”
Overall Location	3		How many vacant or abandoned houses or lots do you currently see in your location?	1-3 range	1=none 2=some 3= a lot
Overall Location	4		How much do you see people fighting or arguing currently in your location?	1-3 range	1=none 2=some 3= a lot
Overall Location	5		How much litter/trash on the streets or sidewalks do you currently see in your location?	1-3 range	1=none 2=Some 3=a lot
Overall Location	6		How much drug use /alcohol use and/or drug dealing do you currently see in your location?	1-3 range	1=none 2=some 3= a lot
Overall Location	7		How safe do you feel in your current environment?	1-3 range	1=not at all safe, 2=somewhat safe, 3=very safe

Overall Location	8		How easy would it be for you to get alcohol if you wanted some where you are right now?	1-3 range	1=not easy at all, 2=somewhat easy, 3=very easy
Overall Location	9		How easy would it be for you to get any drugs/illegal substances if you wanted some where you are right now?	1-3 range	1=not at all, 2=somewhat, 3=very
Overall Location	10		Since your last diary, did you visit a place that: Let you forget your everyday responsibilities, feel relaxed, and lose yourself in your thoughts?	Binary	Yes/No
Overall Location	11		Since your last diary, did you visit a place that: You felt a sense of belonging?	Binary	Yes/No
Overall Location	12		Since your last diary, did you visit a place that: Had beautiful views and/or interesting things to look at?	Binary	Yes/No

Overall Location	13		Since your last diary, did you visit a place that: Had greenery such as plants, flowers, or trees?	Binary	Yes/No
Overall Location	14	If answered no to #10, #11, #12, and #13, then skip #14.	How many minutes did you spend at this place?	Text	Open response
Overall Location	15		Where did you go yesterday? Check all that apply.	Mark all that apply.	“grocery store or other store”, “work”, “friend or family member’s house”, “social outing”, “restaurant, bar, or club”, “exercise or recreation”, “park or another greenspace”, “other”, “didn’t leave house”
Mood Stress	1		Since your last diary entry/last night, how have you felt?	Mark all that apply	“excited”, “angry”, “happy”, “sad”, “stressed”, “tired”, “relaxed”, “bored”, “irritated”, “proud”, “strong”, “scared”, “guilty”, “grateful”, “drunk/high”, “in pain”, “craved alcohol”, “craved other non-prescription drug/substance”, “hungover”, “ill/sick”, “none of the above”
Mood Stress	2		To what degree are your emotions feeling out of control right now?	1-4 range	1=not out of control, 2=somewhat out of control, 3=moderately out of control, 4=very out of control
Mood Stress	3		How much stress are you feeling right now?	1-4 range	1=no stress, 2=a little stress, 3=a moderate amount of stress, 4=a lot of stress
Stigma	1		Since last night/your last diary entry, have you felt like people	Binary	Yes/No

			treated you unfairly?		
Stigma	2	If Yes to Stigma 1	Do you feel that this due to any of the following [check all that apply]?	Mark all that apply	“Your sex or gender”, “your sexual orientation”, “your race or ethnicity”, “your alcohol or drug use”, “your HIV status” "other reason"
Stigma	3		Since last night/your last diary entry, how have you felt about your HIV status?	Mark all that apply	“Proud”, “Happy”, “Stressed”, “Ashamed”, “Not as good as other people”, “Calm”, “Confident”, “Neutral”, “I haven't thought about it”
Substance Use	1		Since last night/your last diary entry, how many drinks containing alcohol have you had?	# of drinks	None<br>1 -2<br>3 -4<br>5-7<br>8+
Substance Use	2		Since last night/your last diary entry, how many times have you used drugs/substances other than alcohol such as marijuana, cocaine, etc.?	# of times	None<br>1 -2<br>3 -4<br>5-7<br>8+
Substance Use	3	If Substance Use 1 or 2 =/0, then show	Who did you drink or get high with?	Mark all that apply	“no one”, “family member”, “friends”, “significant other”, “strangers”, “acquaintances”
Substance Use<br>AM ONLY	1		Please select the substances that you used yesterday.	Mark all that apply	Alcohol; Cannabis/marijuana; Cocaine/crack; Opiates (heroin, methadone, oxycontin, fentanyl); Other non-prescription substances; No substances

Substance Use AM ONLY	2	If anything selected for SU AM ONLY 1	How intoxicated were you yesterday at your peak while using alcohol or any other substance?	1-3 range	1=not at all, 2=some 3=very
Prompt		If anything selected for SU AM ONLY 1	You reported that you drank alcohol or took a drug yesterday. Did you drink or take drugs because:	Mark all that apply.	
Drinking Motives	1	If anything selected for SU AM ONLY 1	My friends or family pressured me to do it	checklist	
Drinking Motives	2	If anything selected for SU AM ONLY 1	It helped me when I felt depressed or nervous	checklist	
Drinking Motives	3	If anything selected for SU AM ONLY 1	It cheered me up when I was in a bad mood	checklist	
Drinking Motives	4	If anything selected for SU AM ONLY 1	It gave me a pleasant feeling	checklist	
Drinking Motives	5	If anything selected for SU AM ONLY 1	It improved a party or celebration	checklist	
Drinking Motives	6	If anything selected for SU AM ONLY 1	It helped me forget about my problems	checklist	
Drinking Motives	7	If anything selected for SU AM ONLY 1	It helped me forget about my health problems	checklist	

Drinking Motives	8	If anything selected for SU AM ONLY 1	None of the above	checklist	
Intent	1		Do you intend to drink any drinks containing alcohol today?	Numeric	Yes, No, I have already drank today
Intent	2		Do you intend to use any drugs/substances such as marijuana, cocaine, etc. today?	Numeric	Yes, No, I have already used today
HIV Specific	1		Did you take your prescribed HIV medication(s) yesterday?	1-3	1=None of my HIV medication<br>2=Some of y HIV medication<br>3=All of my HIV medication
HIV Specific	2		Did you have difficulty fitting your HIV treatment into your daily routine yesterday?	Binary	Yes/No
HIV Specific	3		Were you tempted NOT to take your ART medication yesterday because you were drunk or high?	Binary	Yes/No
HIV Specific	4		Were you tempted NOT to take your ART medication yesterday because you were not feeling well?	Binary	Yes/No

HIV Specific	5		Did your friends encourage you to take your ART meds yesterday?	Binary	Yes/No
HIV Specific	6		Did a partner/family member encourage you to take your ART meds yesterday?	Binary	Yes/No
Prompt			The following is a feeling people might have when managing their HIV medications daily. Please let us know if you were feeling this way yesterday:		
HIV Specific	7		It frustrated me to think that I will have to take these HIV medications every day for the rest of my life.	Binary	Yes/No
HIV Specific	8		I felt in control of this disease by taking my HIV medications as prescribed	Binary	Yes/No
Prompt			Since your last diary, how much stress did you feel		

			from any of the following?		
Stressor/Exposure	1		Being threatened by a stranger or someone you know	1-4	1=no stress / did not experience; 2=a little stress; 3=a moderate amount of stress; 4=a lot of stress
Stressor/Exposure	2		Being hit, kicked, or physically injured or experienced unwanted sexual contact	1-4	1=no stress / did not experience; 2=a little stress; 3=a moderate amount of stress; 4=a lot of stress
Stressor/Exposure	3		Seeing someone get robbed, injured, or threatened.	1-4	1=no stress / did not experience; 2=a little stress; 3=a moderate amount of stress; 4=a lot of stress
Stressor/Exposure	4		Hearing gunfire.	1-4	1=no stress / did not experience; 2=a little stress; 3=a moderate amount of stress; 4=a lot of stress
Stressor/Exposure	5		Experiencing or seeing police violence or intimidation.	1-4	1=no stress / did not experience; 2=a little stress; 3=a moderate amount of stress; 4=a lot of stress
Stressor/Exposure	6		Having an argument with friend, family member, or romantic partner	1-4	1=no stress / did not experience; 2=a little stress; 3=a moderate amount of stress; 4=a lot of stress
Stressor/Exposure	7		Dealing with an illness, injury, or accident	1-4	1=no stress / did not experience; 2=a little stress; 3=a moderate amount of stress; 4=a lot of stress
Stressor/Exposure	8		Having problems with work	1-4	1=no stress / did not experience; 2=a little stress; 3=a moderate amount of stress; 4=a lot of stress
Stressor/Exposure	9		Having a problem with money	1-4	1=no stress / did not experience; 2=a little stress; 3=a moderate amount of stress; 4=a lot of stress

Stressor/Exposure	10		Not feeling supported by friends/family	1-4	1=no stress / did not experience; 2=a little stress; 3=a moderate amount of stress; 4=a lot of stress
Stressor/Exposure	11		Experiencing stress or chaos in my environment	1-4	1=no stress / did not experience; 2=a little stress; 3=a moderate amount of stress; 4=a lot of stress
Stressor/Exposure	12		Dealing with COVID related stress	1-4	1=no stress / did not experience; 2=a little stress; 3=a moderate amount of stress; 4=a lot of stress
Stressor/Exposure	13		Having a transportation problem	1-4	1=no stress / did not experience; 2=a little stress; 3=a moderate amount of stress; 4=a lot of stress
Stressor/Exposure	14		Having to carefully watch what I say and how I say it	1-4	1=no stress / did not experience; 2=a little stress; 3=a moderate amount of stress; 4=a lot of stress
Stressor/Exposure	15		Worrying about running out of food	1-4	1=no stress / did not experience; 2=a little stress; 3=a moderate amount of stress; 4=a lot of stress
Stressor/Exposure	16		Worrying about paying rent or getting evicted	1-4	1=no stress / did not experience; 2=a little stress; 3=a moderate amount of stress; 4=a lot of stress
Stressor/Exposure	17		Worrying about my mental health	1-4	1=no stress / did not experience; 2=a little stress; 3=a moderate amount of stress; 4=a lot of stress
Stressor/Exposure	18		Worrying about my physical health	1-4	1=no stress / did not experience; 2=a little stress; 3=a moderate amount of stress; 4=a lot of stress
Stressor/Exposure	19		Experiencing problems/Issues with health care	1-4	1=no stress / did not experience; 2=a little stress; 3=a moderate amount of stress; 4=a lot of stress
Stressor/Exposure	20		Having stress related to childcare or caregiving	1-4	1=no stress / did not experience; 2=a little stress; 3=a moderate amount of stress; 4=a lot of stress

Stressor/Exposure	21		Other stressful or difficult event, please specify:	1-4	1=no stress / did not experience; 2=a little stress; 3=a moderate amount of stress; 4=a lot of stress
Stressor/Exposure	22	If answered no stressors for stressor/exposure section (#1-21), then skip. Carry forward checked from #1-21.	Which of these stressful experiences bothered you the most?	Multiple choice	Checked answers will move forward to select from
Daily Appraisals	1	If answered no stressors for stressor/exposure section (#1-21), then skip.	How stressful was this event?	1-3 range	1=not at all stressful, 2=somewhat stressful, 3=very stressful
Daily Appraisals	2	If answered no stressors for stressor/exposure section (#1-21), then skip.	How much do you feel you can control the outcome of this event?	1-3 range	1=not at all, 2=somewhat, 3=very much so
Prompt			Sometimes things happen to people that are unusually or especially frightening, horrible, or traumatic. Some examples include: a serious accident or fire, a hurricane or flood, having a loved one die through homicide or suicide, a		

			physical or sexual assault or abuse, or seeing someone be killed or seriously injured. Answer the following questions based on your experiences since your last diary entry in relation to difficult life events:		
PTSD	1		How much were you bothered by disturbing thoughts, activities, or feelings about difficult life events that have happened to you?	1-5 range	1= not at all, 2=a little bit, 3=moderately, 4=quite a bit, 5=extremely
PTSD	2		How much were you bothered by avoiding thoughts, activities, or feelings about difficult life events that have happened to you?	1-5 range	1= not at all, 2=a little bit, 3=moderately, 4=quite a bit, 5=extremely
PTSD	3		How much were you bothered by feeling distant or cut off from other people and/or feeling emotionally numb?	1-5 range	1= not at all, 2=a little bit, 3=moderately, 4=quite a bit, 5=extremely

PTSD	4		How much were you bothered by difficulty concentrating, feeling jumpy or easily startled, feeling overtly alert, or feeling irritable or angry?	1-5 range	1= not at all, 2=a little bit, 3=moderately, 4=quite a bit, 5=extremely
PTSD	5		How much were you bothered by having strong negative beliefs about yourself, other people, or the world (for example, having thoughts such as: I am bad, there is something seriously wrong with me, no one can be trusted, the world is completely dangerous)?	1-5 range	1= not at all, 2=a little bit, 3=moderately, 4=quite a bit, 5=extremely
Sleep Quality	1		How satisfied were you with your sleep last night?	1-3	1= not at all satisfied, 2=somewhat satisfied, 3= very satisfied

AIM III: 6PM DAILY DIARY

Type of Q	Type Q #	Logic	Question	Response Type	Response Options
Overall Location	1		How would you describe your current location overall?	Multiple choice	“workplace”, “inside your home”, “inside another’s home”, “vehicle/bus”, “outside”, “other (please specify)” (write-in option if “other” chosen)
Overall Location	2		Who are you with currently?	Mark all that apply	“no one”, “significant other”, “family member”, “friends”, “acquaintances”, “coworkers”, “strangers”
Overall Location	3		How many vacant or abandoned houses or lots do you currently see in your location?	1-3 range	1=none 2=some 3= a lot
Overall Location	4		How much do you see people fighting or arguing currently in your location?	1-3 range	1=none 2=some 3= a lot
Overall Location	5		How much litter/trash on the streets or sidewalks do you currently see in your location?	1-3 range	1=none 2=Some 3=a lot
Overall Location	6		How much drug use/alcohol use and/or drug dealing do you currently see in your location?	1-3 range	1=none 2=some 3= a lot
Overall Location	7		How safe do you feel in your current environment?	1-3 range	1=not at all safe, 2=somewhat safe, 3=very safe
Overall Location	8		How easy would it be for you to get alcohol if you wanted some where you are right now?	1-3 range	1=not easy at all, 2=somewhat easy, 3=very easy
Overall Location	9		How easy would it be for you to get any drugs/illegal substances if you wanted	1-3 range	1=not at all, 2=somewhat easy, 3=very easy

			some where you are right now?		
Overall Location	10		Since your last diary, did you visit a place that: Let you forget your everyday responsibilities, feel relaxed, and lose yourself in your thoughts?	Binary	Yes/No
Overall Location	11		Since your last diary, did you visit a place that: You felt a sense of belonging?	Binary	Yes/No
Overall Location	12		Since your last diary, did you visit a place that: Had beautiful views and/or interesting things to look at?	Binary	Yes/No
Overall Location	13		Since your last diary, did you visit a place that: Had greenery such as plants, flowers, or trees?	Binary	Yes/No
Overall Location	14	If answered no to #10, #11, #12, and #13, then skip #14.	How many minutes did you spend at this place?	Text	Open response
Mood Stress	1		Since your last diary entry, how have you felt?	Mark all that apply	“excited”, “angry”, “happy”, “sad”, “stressed”, “tired”, “relaxed”, “bored”, “irritated”, “proud”, “strong”, “scared”, “guilty”, “grateful”, “drunk/high”, “in pain”, “craved alcohol”, “craved other non-prescription drug/substance”, “hungover”, “ill/sick”, “none of the above”
Mood Stress	2		To what degree are your emotions feeling out of control right now?	1-4 range	1=not out of control, 2=somewhat out of control 3=very out of control, 4=very out of control

Mood Stress	3		How much stress are you feeling right now?	1-4 range	1=no stress, 2=a little stress 3=a moderate amount of stress, 4=a lot of stress
Stigma	1		Since your last diary entry, have you felt like people treated you unfairly?	Binary	Yes/No
Stigma	2	If Yes to Stigma 1	Do you feel that this due to any of the following [check all that apply]?	Mark all that apply	"Your sex or gender", "your sexual orientation", "your race or ethnicity", "your alcohol or drug use", "your HIV status" "other reason"
Stigma	3		Since your last diary entry, how have you felt about your HIV status?	Mark all that apply	"Proud", "Happy", "Stressed", "Ashamed", "Not as good as other people", "Calm", "Confident", "Neutral", "I haven't thought about it"
Substance Use	1		Since your last diary entry, how many drinks containing alcohol have you had?	# of drinks	None<br>1 -2<br>3 -4<br>5-7<br>8+
Substance Use	2		Since your last diary entry, how many times have you used drugs/substances other than alcohol such as marijuana, cocaine, etc.?	# of times	None<br>1 -2<br>3 -4<br>5-7<br>8+
Substance Use	3	If Substance Use 1 or 2 =/0, then show	Who did you drink or get high with?	Mark all that apply	"no one", "family member", "friends", "significant other", "strangers", "acquaintances"
Prompt			Since your last diary, how much stress did you feel from any of the following?		
Stressor/Exposure	1		Being threatened by a stranger or someone you know	1-4	1=no stress / did not experience; 2=a little stress; 3=a moderate amount of stress; 4=a lot of stress

Stressor/Exposure	2		Being hit, kicked, or physically injured or experienced unwanted sexual contact	1-4	1=no stress / did not experience; 2=a little stress; 3=a moderate amount of stress; 4=a lot of stress
Stressor/Exposure	3		Seeing someone get robbed, injured, or threatened.	1-4	1=no stress / did not experience; 2=a little stress; 3=a moderate amount of stress; 4=a lot of stress
Stressor/Exposure	4		Hearing gunfire.	1-4	1=no stress / did not experience; 2=a little stress; 3=a moderate amount of stress; 4=a lot of stress
Stressor/Exposure	5		Experiencing or seeing police violence or intimidation.	1-4	1=no stress / did not experience; 2=a little stress; 3=a moderate amount of stress; 4=a lot of stress
Stressor/Exposure	6		Having an argument with friend, family member, or romantic partner	1-4	1=no stress / did not experience; 2=a little stress; 3=a moderate amount of stress; 4=a lot of stress
Stressor/Exposure	7		Dealing with an illness, injury, or accident	1-4	1=no stress / did not experience; 2=a little stress; 3=a moderate amount of stress; 4=a lot of stress
Stressor/Exposure	8		Having problems with work	1-4	1=no stress / did not experience; 2=a little stress; 3=a moderate amount of stress; 4=a lot of stress
Stressor/Exposure	9		Having a problem with money	1-4	1=no stress / did not experience; 2=a little stress; 3=a moderate amount of stress; 4=a lot of stress
Stressor/Exposure	10		Not feeling supported by friends/family	1-4	1=no stress / did not experience; 2=a little stress; 3=a moderate amount of stress; 4=a lot of stress
Stressor/Exposure	11		Experiencing stress or chaos in my environment	1-4	1=no stress / did not experience; 2=a little stress; 3=a moderate amount of stress; 4=a lot of stress
Stressor/Exposure	12		Dealing with COVID related stress	1-4	1=no stress / did not experience; 2=a little stress; 3=a moderate amount of stress; 4=a lot of stress

Stressor/Exposure	13		Having a transportation problem	1-4	1=no stress / did not experience; 2=a little stress; 3=a moderate amount of stress; 4=a lot of stress
Stressor/Exposure	14		Having to carefully watch what I say and how I say it	1-4	1=no stress / did not experience; 2=a little stress; 3=a moderate amount of stress; 4=a lot of stress
Stressor/Exposure	15		Worrying about running out of food	1-4	1=no stress / did not experience; 2=a little stress; 3=a moderate amount of stress; 4=a lot of stress
Stressor/Exposure	16		Worrying about paying rent or getting evicted	1-4	1=no stress / did not experience; 2=a little stress; 3=a moderate amount of stress; 4=a lot of stress
Stressor/Exposure	17		Worrying about my mental health	1-4	1=no stress / did not experience; 2=a little stress; 3=a moderate amount of stress; 4=a lot of stress
Stressor/Exposure	18		Worrying about my physical health	1-4	1=no stress / did not experience; 2=a little stress; 3=a moderate amount of stress; 4=a lot of stress
Stressor/Exposure	19		Experiencing problems/Issues with health care	1-4	1=no stress / did not experience; 2=a little stress; 3=a moderate amount of stress; 4=a lot of stress
Stressor/Exposure	20		Having stress related to childcare or caregiving	1-4	1=no stress / did not experience; 2=a little stress; 3=a moderate amount of stress; 4=a lot of stress
Stressor/Exposure	21		Other stressful or difficult event, please specify:	1-4	1=no stress / did not experience; 2=a little stress; 3=a moderate amount of stress; 4=a lot of stress
Stressor/Exposure	22	If answered no stressors for stressor/exposure section (#1-21), then skip. Carry forward checked from #1-21.	Which of these stressful experiences bothered you the most?	Multiple choice	Checked answers will move forward to select from

Daily Appraisals	1	If answered no stressors for stressor/exposure section (#1-21), then skip.	How stressful was this event?	1-3 range	1=not at all stressful, 2=somewhat stressful, 3=very stressful
Daily Appraisals	2	If answered no stressors for stressor/exposure section (#1-21), then skip.	How much do you feel you can control the outcome of this event?	1-3 range	1=not at all, 2=somewhat, 3=very much so
Prompt			Have you done any of the following today to deal with the most stressful experience you had today?	Check all that apply	
Daily Coping	1		Concentrating my efforts on doing something about the situation I'm in	(checklist)	
Daily Coping	2		Getting emotional support from others	(checklist)	
Daily Coping	3		Refusing to believe that it has happened	(checklist)	
Daily Coping	4		Using alcohol or other drugs to help me get through it	(checklist)	
Daily Coping	5		Criticizing myself	(checklist)	
Daily Coping	6		Trying to come up with a strategy about what to do	(checklist)	
Daily Coping	7		Looking for something good in what is happening	(checklist)	
Daily Coping	8		Doing something to think about it less, such as going to movies, watching TV, reading, daydreaming, sleeping, or shopping	(checklist)	

Daily Coping	9		Accepting the reality of the fact that it has happened	(checklist)	
Daily Coping	10		Expressing my negative feelings	(checklist)	
Daily Coping	11		Trying to find comfort in my religion or spiritual beliefs	(checklist)	
Daily Coping	12		Trying to get advice or help from other people about what to do	(checklist)	
Daily Coping	13		Blaming myself for things that happened	(checklist)	
Daily Coping	14		Praying or meditating	(checklist)	
Daily Coping	15		Other (please specify)	(checklist)	
Prompt			Sometimes things happen to people that are unusually or especially frightening, horrible, or traumatic. Some examples include: a serious accident or fire, a hurricane or flood, having a loved one die through homicide or suicide, a physical or sexual assault or abuse, or seeing someone be killed or seriously injured. Answer the following questions based on your experiences since your last diary entry in relation to difficult life events:		
PTSD	1		How much were you bothered by disturbing thoughts, activities, or feelings about difficult life	1-5 range	1= not at all, 2=a little bit, 3=moderately, 4=quite a bit, 5=extremely

			events that have happened to you?		
PTSD	2		How much were you bothered by avoiding thoughts, activities, or feelings about difficult life events that have happened to you?	1-5 range	1= not at all, 2=a little bit, 3=moderately, 4=quite a bit, 5=extremely
PTSD	3		How much were you bothered by feeling distant or cut off from other people and/or feeling emotionally numb?	1-5 range	1= not at all, 2=a little bit, 3=moderately, 4=quite a bit, 5=extremely
PTSD	4		How much were you bothered by difficulty concentrating, feeling jumpy or easily startled, feeling overtly alert, or feeling irritable or angry?	1-5 range	1= not at all, 2=a little bit, 3=moderately, 4=quite a bit, 5=extremely
PTSD	5		How much were you bothered by having strong negative beliefs about yourself, other people, or the world (for example, having thoughts such as: I am bad, there is something seriously wrong with me, no one can be trusted, the world is completely dangerous)?	1-5 range	1= not at all, 2=a little bit, 3=moderately, 4=quite a bit, 5=extremely
Prompt			How much did you believe these statements to be true today?		

Post Trauma Cognitions	1		I am a good person	Binary	Yes/No
Post Trauma Cognitions	2		I don't trust anyone anymore	Binary	Yes/No
Post Trauma Cognitions	3		I trust my own judgement	Binary	Yes/No
Post Trauma Cognitions	4		I feel as though I can depend on other people	Binary	Yes/No
Post Trauma Cognitions	5		Most people are basically caring	Binary	Yes/No
Prompt			How often did you use the following strategies to deal with stress today?		
Emotion Regulation	1		I thought over and over again about my emotions	1-3 range	1= Not at all 2=Sometimes 3=A lot of the time
Emotion Regulation	2		I was careful not to express my emotions to others	1-3 range	1= Not at all 2=Sometimes 3=A lot of the time
Emotion Regulation	3		I accepted my emotions as valid and important	1-3 range	1= Not at all 2=Sometimes 3=A lot of the time
Emotion Regulation	4		I ignored my emotions	1-3 range	1= Not at all 2=Sometimes 3=A lot of the time
Physical Activity	1		What type of physical activity did you do today, if any?	1-3 range	1=None, 2= moderate physical activity or walking that increases your heart rate or makes you breathe harder than normal? (Ex. Bicycling at a regular pace, carrying light loads, mowing the lawn), 3= vigorous physical activity that makes you sweat or breathe/pant heavily? (Ex. Jogging, heavy lifting, fast bicycling, aerobics), 4=other
COVID	1		Did the novel coronavirus or covid-19 affect your ability	Binary	Yes/No.

			to socialize today, such as seeing friends or going out?		
COVID	2		Did the novel coronavirus or covid-19 affect your movement, such as where you went or how you got around?	Binary	Yes/No.
Positive Social Interaction	1		How many social interactions (in person, over the phone, or electronically) did you have today that made you feel good or happy?	0-4 range	0=None, 1=1-2, 2=3-5, 3=6-9, 4=10 or more
Motivation	1		What if anything motivated you today to take care of your health?	Text	Open response
Skill Check	1		Did you use any of the following learned skills today, either within this app or in your daily life?	Check all that apply	“Breathwork”, “Journaling”, “Practicing coping skills”, “Goal setting”, “Challenging negative thoughts”, “Mindfulness”
